# Productive Parvovirus B19 Infection of Primary Human Erythroid Progenitor Cells at Hypoxia Is Regulated by STAT5A and MEK Signaling but not HIFα

**DOI:** 10.1371/journal.ppat.1002088

**Published:** 2011-06-16

**Authors:** Aaron Yun Chen, Steve Kleiboeker, Jianming Qiu

**Affiliations:** 1 Department of Microbiology, Molecular Genetics and Immunology, University of Kansas Medical Center, Kansas City, Kansas, United States of America; 2 ViraCor-IBT Laboratories, Lee's Summit, Missouri, United States of America; Cornell University, United States America

## Abstract

Human parvovirus B19 (B19V) causes a variety of human diseases. Disease outcomes of bone marrow failure in patients with high turnover of red blood cells and immunocompromised conditions, and fetal hydrops in pregnant women are resulted from the targeting and destruction of specifically erythroid progenitors of the human bone marrow by B19V. Although the *ex vivo* expanded erythroid progenitor cells recently used for studies of B19V infection are highly permissive, they produce progeny viruses inefficiently. In the current study, we aimed to identify the mechanism that underlies productive B19V infection of erythroid progenitor cells cultured in a physiologically relevant environment. Here, we demonstrate an effective reverse genetic system of B19V, and that B19V infection of *ex vivo* expanded erythroid progenitor cells at 1% O_2_ (hypoxia) produces progeny viruses continuously and efficiently at a level of approximately 10 times higher than that seen in the context of normoxia. With regard to mechanism, we show that hypoxia promotes replication of the B19V genome within the nucleus, and that this is independent of the canonical PHD/HIFα pathway, but dependent on STAT5A and MEK/ERK signaling. We further show that simultaneous upregulation of STAT5A signaling and down-regulation of MEK/ERK signaling boosts the level of B19V infection in erythroid progenitor cells under normoxia to that in cells under hypoxia. We conclude that B19V infection of *ex vivo* expanded erythroid progenitor cells at hypoxia closely mimics native infection of erythroid progenitors in human bone marrow, maintains erythroid progenitors at a stage conducive to efficient production of progeny viruses, and is regulated by the STAT5A and MEK/ERK pathways.

## Author Summary

Human parvovirus B19 (B19V) is the etiological agent of fifth disease seen in children, aplastic crisis in sickle cell disease patients, chronic anemia in immunocompromised patients, and hydrops fetalis in pregnant women. After more than 35 years since its discovery, B19V was still unable to be propagated *in vitro* in a productive and sustainable manner, which directly delayed the progress of B19V pathogenesis study and consequently finding ways to treat patients infected with B19V. We cultured human erythroid progenitor cells at a hypoxic condition by mimicking the natural niches of human bone marrow. Our current work reveals, for the first time, a long-term B19V infection of *ex vivo* expanded erythroid progenitor cells at hypoxia. Thus, this finding will largely facilitate the study of the mechanisms underlying B19V infection and more importantly identification of approaches to treat B19V infection. Finally, the identification of the cellular signaling pathways in regulating B19V replication sheds light on the virus-host interaction and will nominate potential candidates for anti-virus drug targeting.

## Introduction

Human parvovirus B19 (B19V) is the only parvovirus so far confirmed to be pathogenic to humans [1]. Infection by this virus is the cause of the highly contagious “fifth disease” in children. It can also result in serious, and occasionally fatal, hematologic diseases in susceptible patients. Acute B19V infection can cause transient aplastic crisis in patients with high levels of red blood cell destruction and erythrocyte turnover (e.g., sickle-cell disease patients). Pure red-cell aplasia and chronic anemia can also be a manifestation of persistent B19V infection in immunocompromised patients [2]. Finally, aplastic crisis in the fetus and hydrops fetalis can occur as a result of infection-induced anemia in pregnant women [3].

B19V belongs to the genus *Erythrovirus* of the *Parvoviridae* family [4]. Infection by B19V is restricted exclusively to human erythroid progenitor cells (EPCs) at the stage from the late burst-forming unit-erythroid (BFU-E) to colony-forming unit-erythroid (CFU-E) [2], [5]. The 5.6-kb linear single-stranded DNA genome (ssDNA), which is flanked by two identical terminal hairpin repeats, encodes nonstructural proteins (NS1, 11 kDa and 7.5 kDa) and two capsid proteins (VP1 and VP2) [6], [7]. A model of “rolling hairpin”-dependent DNA replication of B19V has been proposed [8]. However, neither the mechanism underlying the unique tropism of B19V genome replication in human EPCs, nor the cellular factors involved, have been identified.

Currently, the study of B19V infection is hampered by the lack of both suitable animal models and an efficient and productive *in vitro* B19V propagation system. Attempts have been made to use *in vitro* cultures of human bone marrow cells [9], megakaryoblastoid cells (the UT7/Epo cell line [10] and its subclone UT7/Epo-S1 [11]), and erythroid leukemia cells (the KU812Ep6 cell line [12]). However, all of these are merely semi-permissive to B19V infection, producing only low levels of viral proteins and viral genomes [13], [14]. Recently, *ex vivo* expanded primary human EPCs infected with B19V [15]–[17] have shown extraordinary promise as an alternative system, based on high expression of B19V capsid and non-structural proteins. Nevertheless, these expanded EPCs failed to produce infectious virus at levels sufficient to maintain the virus titer, and thus do not constitute a productive culture system. Notably, B19V infection of primary EPCs at low oxygen (1% O_2_, hypoxia) has been shown to promote B19V infection, an effect that has been proposed to be as an outcome of enhanced stimulation of the P6 promoter by HIF1α [18], a key transcription factor that is stabilized in the context of hypoxia [19].

The extreme tropism of B19V for human erythroid progenitors of the bone marrow and the high viremia [up to 3×10^13^ genomic copies per ml of plasma [13]] characteristic of B19V-infected patients remain puzzling. The B19V receptor (Globoside) [20] and its co-receptors (CD49e and KU80) [21], [22] have been invoked based on their involvement in virus entry, but only partially account for B19V tropism [23], [24]. Erythropoietin (Epo) is another candidate, as it is required for the maintenance and permissiveness of human EPCs to B19V infection [10]–[12], [16]. Using *ex vivo* expanded EPCs, we recently demonstrated that signaling by Epo and the Epo receptor (EpoR) serves as a molecular switch for B19V DNA replication in cells which have internalized the virus [24]. However, how Epo/EpoR signaling influences B19V replication and which downstream molecules facilitate viral DNA replication have not been understood. The EpoR signaling pathway is triggered by ligation of Epo to EpoR, which activates Janus kinase 2 (Jak2) such that it phosphorylates both itself and EpoR at multiple tyrosine sites [25]. This initiates a kinase cascade with three major branches, starting at the signal transducer and activator of transcription 5A (STAT5A), mitogen-activated protein kinase (ERK/MAPK) kinase (MEK) and phosphatidylinositol-3 kinase (PI3K). The balance of these three signaling pathways directs the differentiation and proliferation of erythroid progenitors to erythrocytes.

In the present study, we report that *ex vivo* expanded CD36^+^ EPCs cultured in the context of hypoxia support sustained productive B19V infection, efficiently amplifying the numbers of infectious virions within the cell by enhancing the level of viral DNA replication. Our results reveal that STAT5A is critical in promoting hypoxia-facilitated B19V infection in CD36^+^ EPCs during erythropoiesis, whereas MEK negatively regulates B19V infection.

## Results

### Hypoxia promotes productive and sustainable B19V infection of CD36^+^ EPCs

**Figure 1:**
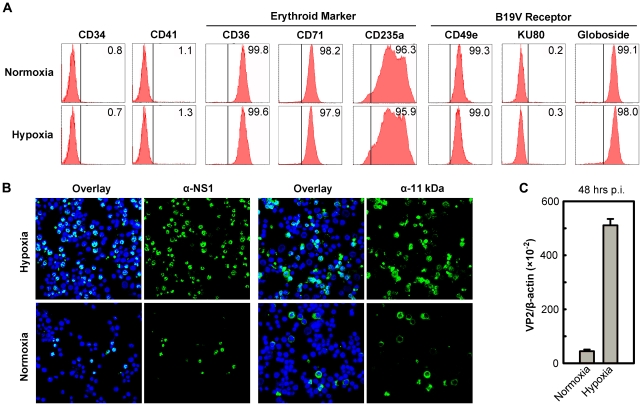
Support of B19V infection of CD36^+^ EPCs under hypoxia *vs.* normoxia. (**A**) Status of CD36^+^ EPCs cultured under hypoxia *vs.* normoxia. Day 4 HSCs were *ex vivo* expanded under either hypoxia or normoxia to produce CD36^+^ EPCs. At day 8, the cells were profiled by flow cytometry analysis for surface antigens indicated. Numbers shown in each plot are percentage of positive cells. (**B&C**) B19V infection under hypoxia *vs.* normoxia. Day 8 CD36^+^ EPCs were infected with B19V at an MOI of 5,000 gc/cell. (**B**) At 48 hrs p.i., immunofluorescence staining was performed using anti-NS1 or anti-11 kDa antiserum. DAPI was used to stain the nucleus. Confocal images were taken at a magnification of 40×(objective lens) using an Eclipse C1 Plus confocal microscope controlled by EZ-C1 software (Nikon). (**C**) At 48 hrs p.i., mRNA was extracted from infected cells, and the levels of B19V VP2-encoding mRNA per β-actin mRNA were quantified.

To best mimic the bone marrow microenvironment in which EPCs are found *in vivo*, we cultured a day 4 stock of CD34^+^ hematopoietic stem cells (HSCs), that has been cultured under normoxia from day 0 for a further 4 days under either normoxia (21% O_2_) or hypoxia (1% O_2_). The day 8 CD36^+^ EPCs produced under the two conditions were confirmed to be nearly identical with respect to the profiles of the major cell surface erythroid markers (CD36, CD71 and CD235a) [24], the B19V receptor (Globoside) [20] and the B19V co-receptors (CD49e and KU80) [21], [22] (Figure 1A). The absence of both CD34 (hematopoietic maker) and CD41 (megakaryoblastic maker), and the nearly complete expression of CD36, CD71 (Transferrin receptor) and CD235a (Glycophorin A), on the cell surface under each condition indicates that both sets of CD36^+^ EPCs produced fall into a range of late BFU-E to CFU-E progenitors (Figure 1A). Moreover, the levels of B19V receptor and co-receptors on the cell surface were similar on CD36^+^ EPCs cultured under the two conditions.

We next infected both sets of CD36^+^ EPCs with B19V at a multiplicity of infection (MOI) of 5,000 genome copies (gc)/cell. At 48 hrs postinfection (p.i.), the virus-encoded nonstructural proteins NS1 and 11 kDa were detected in more than 70% of infected cells that had been cultured under hypoxia, but in fewer than 25% of infected cells that had been cultured under normoxia (Figure 1B). Consistent with this finding, the level of VP2-encoding mRNA in infected cells cultured under hypoxia was 10 times greater than that detected in the cells cultured under normoxia (Figure 1C). Similar results were obtained when the B19V infection of day 8 CD36^+^ EPCs that had been cultured under hypoxia starting at day 0 was examined (data not shown). The CD36^+^ EPCs that were switched to hypoxia at day 4 of culture were therefore used for the remainder of this study. Additionally, the efficiency of the B19V infection of the B19V semi-permissive UT7/Epo-S1 cells also increased drastically under hypoxia (Figure S1A).

**Figure 2:**
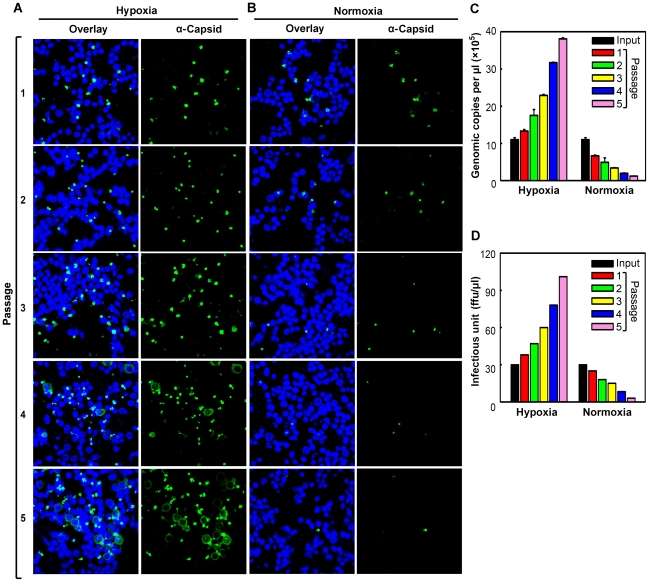
Production of progeny virus in CD36^+^ EPCs under hypoxia *vs.* normoxia. Day 8 CD36^+^ EPCs were infected with B19V at a low MOI (1,000 gc/cell) under either hypoxia or normoxia. At 72 hrs p.i., infected cells were lysed by repeated freeze-thaw cycles, to release progeny virus. After a brief spin, one third of the lysate from each culture was used to infect fresh day 8 CD36^+^ EPCs cultured under the same conditions. (**A&B**) At each passage, infected cells were examined by immunofluorescence to detect staining with an anti-B19V capsid antibody (detection for assembled capsids). (**C&D**)The production of progeny virus was quantified by qPCR-based assessment of the number of copies of the viral genome (C) and assessed for infectious units by titration of ffu (D).

In order to examine the productivity and sustainability of B19V infection of CD36^+^ EPCs under hypoxia, we passaged B19V preparations harvested from the initial infections under both hypoxia and normoxia, through CD36^+^ EPCs cultured under each condition. Our results showed that, at the same MOI, B19V infection generated approximately (∼)5 times more capsid-expressing cells among cells infected in the context of hypoxia than in those infected in the context of normoxia (Passage 1) (Figure 2A). Surprisingly, when cells were maintained under hypoxia, the number of capsid-expressing cells increased as the virus was passaged, reaching a rate of ∼75% infection by the fifth passage. In contrast, when the cells were maintained under normoxia, the number of capsid-expressing cells decreased, with fewer than 5% positive for capsid protein by the fifth passage (Figure 2B). This difference was consistent with the progeny virus yields under the two conditions, as assessed by titration of infectious units (ffu) and virus particles (gc) (Figure 2C&D, respectively). Specifically, at the fifth passage, a virus yield of over 90 ffu/µl was obtained from infected cells cultured under hypoxia, whereas a yield of less than 5 ffu/µl was obtained from infected cells cultured under normoxia. These results demonstrate that B19V infection of CD36^+^ EPCs under hypoxia leads to a sustainable “productive infection,” and that in contrast, B19V infection of cells under normoxia leads to only “permissive infection,” with inefficient production of progeny virus resulting in aborted infection after several passages.

### Hypoxia facilitates B19V DNA replication, but not virus entry or intracellular trafficking

**Figure 3:**
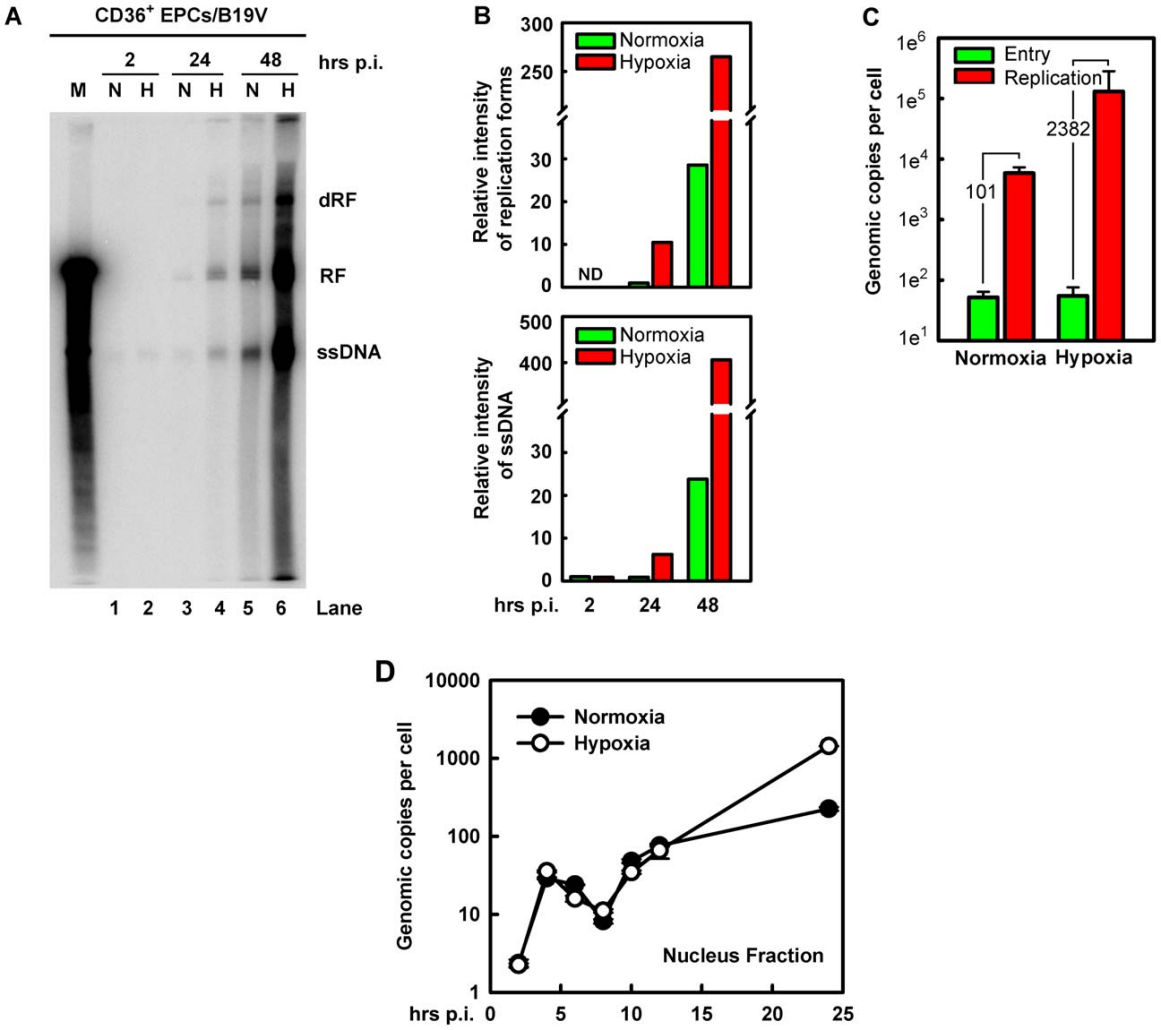
B19V DNA replication, virus entry and intracellular trafficking in CD36^+^ EPCs under hypoxia *vs.* normoxia. B19V DNA replication, entry and trafficking were assessed in day 8 CD36^+^ EPCs infected with B19V at an MOI of 5,000 gc/cell under each condition. (**A&B**) At the indicated times p.i., Hirt DNA was extracted and analyzed by Southern blotting. (A) The full blot is shown. M, marker, a 5.6-kb B19V DNA; N, normoxia; H, hypoxia; RF, replicative form DNA; dRF: double replicative form DNA; ssDNA: single-stranded DNA. (B) The bands of the viral RF DNA and ssDNA genome in the blot were quantified and values shown are relative to the normoxia-cultured sample of either collected at 24 hrs p.i. (upper panel) or at 2 hrs p.i. (lower panel). (**C**) B19V entry into the cell and virus replication were assessed. The entered number of viral genomes within the cell under each condition was assessed, and is shown in green bars. Duplicated sets of treated cells were maintained under the respective condition until 48 hrs p.i., at which point total viral DNA was quantified. Results are shown as absolute number of genomic copies per cell in red bars. (**D**) The rate of B19V trafficking to the nucleus and replication was measured in infected cells, at the indicated time points. The numbers of genome copies per cell in the nucleus are shown.

We next examined which step during the virus life cycle is facilitated in B19V-infected CD36^+^ EPCs cultured under hypoxia. To this end, we infected CD36^+^ EPCs with B19V under each condition. At 2 hrs p.i, we observed an equivalent level of virus bound to the cells under the two conditions (Figure 3A, compare lanes 1 and 2). However, at 24 hrs p.i., we detected an ∼5-fold increase in both the replicative form of double-stranded DNA (RF DNA) and the single stranded DNA (ssDNA) genome in B19V-infeceted CD36^+^ EPCs under hypoxia (Figure 3A&B, 24 hrs). At 48 hrs p.i, an ∼10-fold increase of the RF DNA form and an ∼20-fold increase of the ssDNA viral genome were observed in the cells cultured under hypoxia (Figure 3A&B, 48 hrs). Notably, the ratio of B19V ssDNA to RF DNA did not differ significantly in the two groups of infected cells (∼1∶1; Figure 3A, lanes 5 and 6). These results suggest that hypoxia promotes replication of the B19V DNA as well as production of B19V progeny viruses (by ∼20-fold in the level of ssDNA viral genome).

This notion was confirmed by a careful assessment of virus entry by a qPCR-based B19V DNA replication assay (Figure 3C), in which cells at 2 hrs p.i. were pretreated with trypsin to remove attached virion, and the number of viral genomes within the cells was quantified. No significant difference in virus entry was observed for CD36^+^ EPCs cultured under normoxia *vs.* hypoxia, suggesting that hypoxia does not affect viral entry. This result is consistent with the fact that hypoxia did not affect the levels of the B19V receptor and co-receptors on cell surface (Figure 1A). In spite of the equivalent numbers observed for virus entry at 2 hrs p.i., the number of B19V genome copies at 48 hrs p.i., was increased by 2,382-fold in B19V-infected CD36^+^ EPCs cultured under hypoxia but only 101-fold in the cells cultured under normoxia, thus replication of the B19V DNA is 20-fold more efficient in cells cultured under hypoxia than in cells cultured under normoxia (Figure 3C).

To rule out the possibility that the enhanced viral DNA replication in CD36^+^ EPCs cultured under hypoxia was due to an increase in intracellular virus trafficking after virus had entered the cell, we examined number of viral genomes in the nucleus during early infection. As shown in Figure 3D, at 2 hrs p.i. the level of viral genomes in the nucleus was next to undetectable in both sets of CD36^+^ EPCs. Viral genome accumulation began abruptly at 4 hrs p.i., and decreased gradually until 8 hrs p.i., presumably as the result of virus clearance by the host. Interestingly, the viral genomes in nuclei increased again sharply from 8 to 10 hrs p.i., indicating replication of the viral genome. Dramatic differences in the number of the viral genomes in the nuclear fractions from normoxia- and hypoxia-cultured EPCs were not observed until after 12 hrs p.i., at which point the levels of the viral genome in the nuclei of hypoxia-cultured CD36^+^ EPCs began to rise relative to those in normoxia-cultured CD36^+^ EPCs, reaching a level ∼6.4 times higher by 24 hrs p.i. Overall, during early infection (2–12 hrs p.i.), there was no significant difference in levels of viral genome in the nucleus of infected cells cultured under hypoxia *vs.* normoxia, suggesting that the B19V genome that has entered the cells was transported to the nucleus at a similar rate under the two conditions, and that the difference in viral genome number at later stages of infection was likely due to increased viral DNA replication in cells cultured under hypoxia.

**Figure 4:**
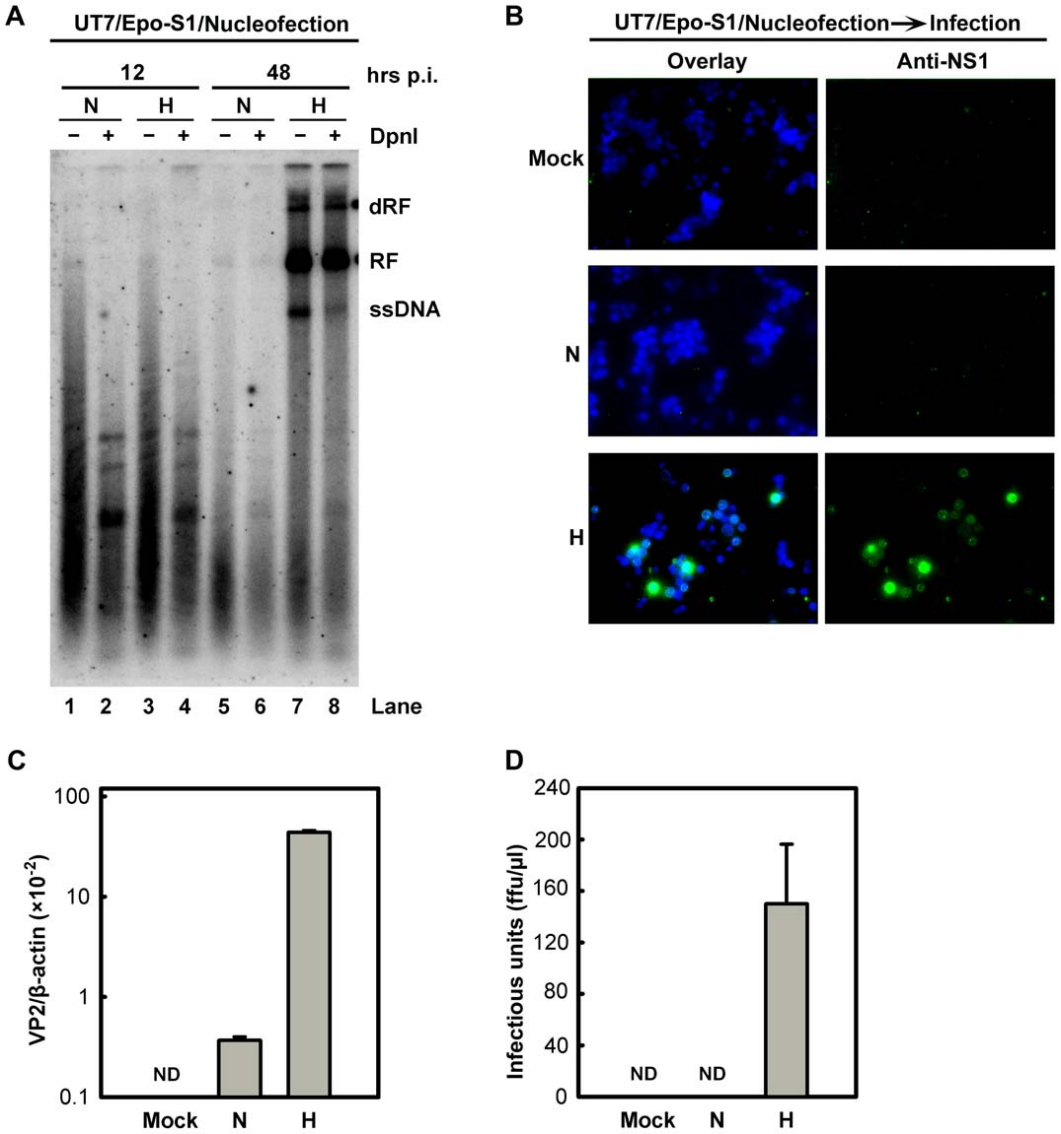
B19V DNA replication and *de novo* production of infectious virions in UT7/Epo-S1 cells under hypoxia. (**A**) The rate of replication of transfected B19V infectious DNA (M20) was measured in UT7/Epo-S1 cells under hypoxia (H) *vs.* normoxia (N). UT7/Epo-S1 cells were pre-cultured under hypoxia for 2 days prior to nucleofection with the M20 DNA. At the times indicated, Hirt DNA was prepared, digested with (+) or without (−) Dpn I, and analyzed by Southern blotting. dRF, RF and ssDNA bands are indicated. (**B, C&D**) B19V infectious virions produced from nucleofection of the M20 DNA into UT7/Epo-S1 cells was boosted under hypoxia. UT7/Epo-S1 cells cultured under hypoxia (H) or normoxia (N) were nucleofected with the M20 DNA. At 48 hrs post-nucleofection, the cells were pelleted and resuspended in CD36^+^ EPC expansion medium followed by three cycles of freeze-thaw and a briefly spin. This virus preparation was used to infect hypoxia-cultured CD36^+^ EPCs. At 48 hrs p.i., immunofluorescence staining was performed using anti-NS1 (B), images were acquired at a magnification of 20×(objective lens) using an Eclipse Ti-S inverted microscope (Nikon) controlled by MetaMorph software (Molecular Devices); and the levels of VP2-encoding mRNA per β-actin mRNA were quantified (C). Yields of infectious virions in ffu were calculated (D). ND, not detectable.

We also tested the absolute effects of hypoxia on B19V DNA replication, in cells transfected with a B19V infectious DNA (M20) [26]. Since CD36^+^ EPCs are known to be difficult to transfect, with the only somewhat successful method (nucleofection) causing a high rate of cell death [27], we instead transfected the B19V-semipermissive UT7/Epo-S1 cells. The M20 infectious DNA has been shown to replicate in UT7/Epo-S1 cells, though very poorly [26], [28]. Surprisingly, the M20 DNA replicated efficiently in cells cultured under hypoxia, at a rate of ∼78 times higher than in cells cultured under normoxia (Figure 4A, compare lanes 6 and 8), and the ssDNA viral genome was clearly detected in transfected cells cultured under hypoxia (Figure 4A, compare lanes 5 and 7). More importantly, infectious virions were produced efficiently at a level of ∼150 ffu per µl from M20-transfected UT7/Epo-S1 cells cultured under hypoxia, but not from the counterparts cultured under normoxia (Figure 4B–D).

Taken together, these results show that growth in the context of hypoxia facilitates B19V infection at the stage of viral DNA replication within the nucleus, rather than promoting viral entry, intracellular trafficking through the cytoplasm, or packaging of the ssDNA viral genome. Moreover, our results provide a practical approach to generate sufficient B19V progeny virions from the infectious clone for genetic studies in the future.

### B19V infection of hypoxia-cultured CD36^+^ EPCs is not facilitated by hypoxia-inducible factor α (HIFα)

**Figure 5:**
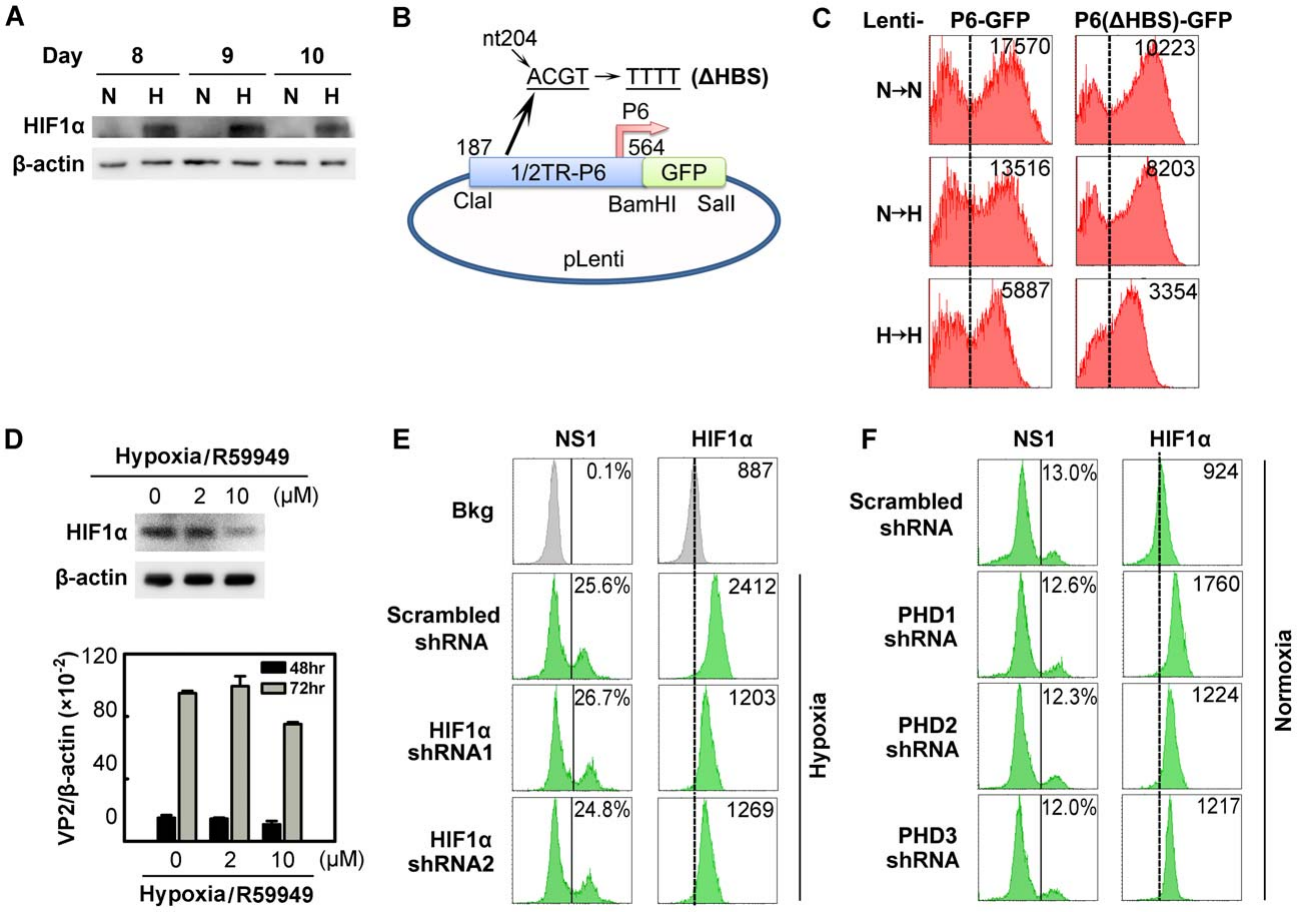
Regulation of B19V infection of CD36^+^ EPCs by HIFα under hypoxia *vs.* normoxia. (**A**) HIF1α levels in CD36^+^ EPCs cultured under normoxia (N) or hypoxia (H). Cell lysates were prepared on the indicated days and were analyzed by Western blotting with anti-HIF1α and anti-β-actin. (**B&C**) Effects of the putative HIF-HBS in the P6 promoter. Day 7 hypoxia- or normoxia-cultured CD36^+^ EPCs were transduced with Lenti-P6-GFP or Lenti-P6(ΔHBS)-GFP (B), and then were either maintained under normoxia (→N) or hypoxia (→H). At 48 hrs post-transduction, level of GFP expression (as MFI) was measured by flow cytometry. (**D**) Effects of R59949 on B19V infection. Day 8 hypoxia-cultured CD36^+^ EPCs were treated with R59949 at the final concentrations shown. At 24 hrs post-treatment, cells were collected for HIF1α detection by Western blotting, or infected with B19V at an MOI of 1,000 gc/cell. At 48 and 72 hrs p.i., the levels of VP2-encoding mRNA per β-actin mRNA were quantified. (**E&F**) Effects of HIF1α and PHD knockdown on B19V infection. Day 7 CD36^+^ EPCs cultured under each condition were transduced with the indicated lentiviruses. At 48 hrs post-transduction, cells were infected with B19V (at MOIs of 2,000 and 4,000 gc/cell for hypoxia- and normoxia-cultured cells, respectively). At 48 hrs p.i., the expression levels of NS1 (as % of positive cells) and HIF1α (as MFI) were analyzed by flow cytometry in lentivirus-transduced (GFP^+^) cells. Dashed reference lines were selected arbitrarily to show the relative position of the peaks, and Bkg (background) represents the secondary antibody only control.

We next sought to identify the cellular signaling pathways that contribute to the increased B19V DNA replication observed in CD36^+^ EPCs under hypoxia. HIF1α is a transcription factor that is the key initiator upregulated in cells under hypoxia [29], [30], and it has been shown to interact with a putative HIF-binding site (HBS) in the B19V P6 promoter [18]. As expected, the level of HIF1α was elevated in hypoxia-cultured CD36^+^ EPCs (Figure 5A). To examine whether hypoxia has an effect on B19V transcription from the P6 promoter, we generated a lentivirus that bears a GFP expression cassette driven either by the B19V P6 promoter (Lenti-P6-GFP) or by a mutant P6 promoter in which the HBS has been knocked out [Lenti-P6(ΔHBS)-GFP] [18] (Figure 5B). As shown in Figure 5C, the mean fluorescence intensity (MFI) values for both Lenti-P6-GFP- and Lenti-P6(ΔHBS)-GFP-transduced cells were decreased in the cells cultured under hypoxia, by ∼20% (Figure 5C, compare panels N→N to N→H), indicating that the wild-type and mutant P6-promoters do not differ in their response to hypoxia or in their response to the stabilized HIF1α. However, we observed that the MFI values of the Lenti-P6(ΔHBS)-GFP-transduced cells were consistently lower than those of the Lenti-P6-GFP-transduced cells under both culture conditions (Figure 5C). These findings imply that transcription factors other than HIF1α may bind to the region spanning the 5′ACGT3′ sequence of the P6 promoter. We also transfected a CMV promoter-driven P6-GFP into UT7/Epo-S1 cells and examined the GFP expression in response to HIF1α that was stabilized by CoCl_2_ [31] (Figure S1C–F). The stabilized HIF1α expression failed to alter GFP expression in transfected cells, consistent with the observation that B19V P6 promoter activity does not respond to the level of HIF1α expression in CD36^+^ EPCs cultured under hypoxia.

We next evaluated the effect of stabilized HIF1/2α on B19V replication in cells under hypoxia, using a pharmacological inhibitor of diacylglycerol kinase (R59949), which decreases the level of HIF1/2α by activating HIF prolylhydroxylases (PHD) [32] and proteasome-dependent degradation of hydroxylated HIFα [19]. Application of 2 µM R59949 resulted in ∼30% inhibition of HIF1α expression, but the B19V VP2-encoding mRNA remained at the same level as in the control (Figure 5D). At 10 µM R59949, HIF1α was inhibited by more than 60%, and the viral VP2-encoding mRNA level decreased slightly (Figure 5D), possibly as a result of the known gentle cytotoxicity of the inhibitor (Figure S4A). This result supports the notion that HIF1/2α does not facilitate B19V infection in CD36^+^ EPCs cultured under hypoxia.

We also employed lentiviruses expressing HIF1α-specific small hairpin RNAs (shRNAs) to test the effects of HIF1α knockdown on B19V infection. Although HIF1α shRNA-expressing lentiviruses decreased HIF1α expression by ∼70% compared to the scrambled shRNA-expressing control lentivirus, they failed to alter the number of cells expressing NS1 at detectable levels (Figure 5E). In addition, the expression of HIF2α- and HIF3α-specifc shRNAs had no significant effects on B19V infection of CD36^+^ EPCs under hypoxia (Figure S2). We also applied shRNAs targeting three isoforms of HIF PHD to promote HIFα expression in CD36^+^ EPCs cells cultured under normoxia. Although moderate increases in HIF1α expression were achieved in HIF PHD1-3 shRNA-transduced cells under normoxia, none of the three shRNAs affected the number of NS1-expressing cells among transduced (GFP^+^) cells (Figure 5F).

Collectively, these results demonstrate that HIFα stabilized (by either hypoxia or PHD inhibition) does not contribute to the increased efficiency of B19V infection of CD36^+^ EPCs cultured under hypoxia.

### Hypoxia reduces the differentiation potential and the proliferation rate of CD36^+^ EPCs

**Figure 6:**
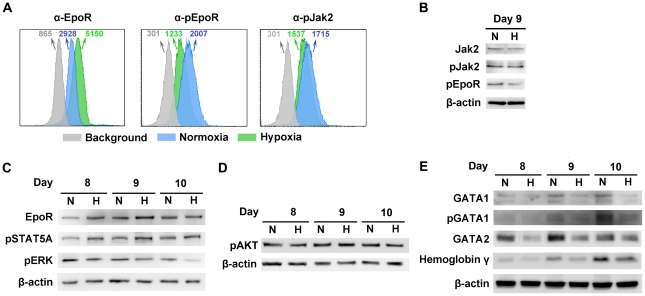
Characterization of CD36^+^ EPCs cultured under normoxia *vs.* hypoxia. Day 4 HSCs were expanded under either hypoxia (H) or normoxia (N). (**A**) At day 9, cell surface EpoR, intracellular pEpoR and pJak2 were analyzed by flow cytometry. Numbers shown are MFI. (**B, C&D**) On the days indicated, the cells were analyzed for the expression of proteins by Western blotting. (**E**) At the days indicated, Western blotting was used to assess the levels of intracellular markers of erythroid differentiation, including GATA1 (total and phosphorylated), GATA2 and hemoglobin-γ.

As Epo/EpoR signaling is critical to B19V replication [24], we hypothesized that CD36^+^ EPCs may undergo changes in Epo/EpoR signaling under hypoxia. To test this hypothesis, we examined the levels of EpoR on cell surface and the phosphorylation status of both EpoR and Jak2 in CD36^+^ EPCs under both conditions. We found that at day 9 EpoR was increased ∼2-fold on the cell surface of CD36^+^ EPCs under hypoxia (Figure 6A), but that the level of phosphorylated EpoR (pEpoR) was decreased by ∼40% in cells under hypoxia (Figure 6A&B). Nevertheless, Jak2 phosphorylation (pJak2) was similar in cells cultured under the two conditions (Figure 6A&B). These findings led us to further examine the signaling pathways downstream of Epo/EpoR signaling. As shown in Figure 6C, the total levels of cellular EpoR were elevated in cells cultured under hypoxia, consistent with the increase in cell surface expression of EpoR (Figure 6A). Strikingly, phosphorylated STAT5A (pSTAT5A), the major outcome of Epo/EpoR signaling and a key driver of erythropoiesis [25], was elevated significantly in cells cultured under hypoxia, whereas phosphorylated ERK (pERK), which is critical for the proliferation and survival of erythroid progenitors [33], [34], was clearly decreased in cells cultured under hypoxia (Figure 6C). In line with these observations, CD36^+^ EPCs proliferated more slowly under hypoxia than under normoxia, as evidenced by both cell counts and an ATP-based cell proliferation assay (Text S1 and Figure S3A&B). Consistent with this finding, the percentage of S-phase cells among normoxia-cultured CD36^+^ EPCs was higher on average than that among their hypoxia-cultured counterparts, and the level of the sub-G0 population of CD36^+^ EPCs under hypoxia lagged behind that of CD36^+^ EPCs under normoxia (Figure S3C). We also examined the PI3K/AKT pathway of CD36^+^ EPCs under hypoxia. Phosphorylated AKT (pAKT) remained at a similar level in cells cultured under the two conditions (Figure 6D), suggesting that AKT may not play an important role in the enhancement of B19V infection of CD36^+^ EPCs in the context of hypoxia.

We further probed the differentiation status of these cells with respect to intracellular makers of erythroid differentiation [35]. These include GATA1, GATA2 and hemoglobin-γ. We found that the levels of GATA1, GATA2 and hemoglobin-γ, as well as phosphorylation of the GATA1 were higher in normoxia-cultured CD36^+^ EPCs than in their hypoxia-cultured counterparts (Figure 6E), indicating that the normoxia-cultured CD36^+^ EPCs are likely more differentiated.

### B19V infection is facilitated by phosphorylated STAT5A

**Figure 7:**
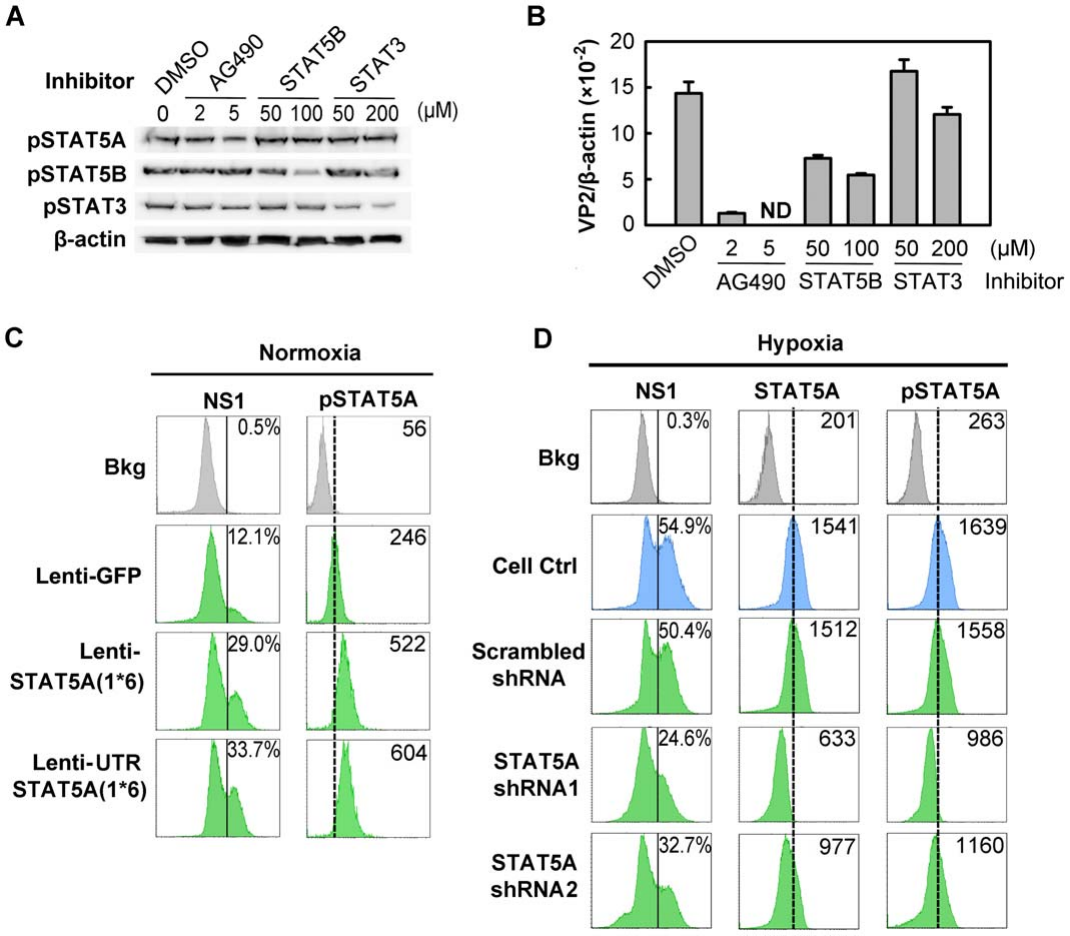
Regulation of B19V infection of CD36^+^ EPCs by phosphorylated STAT5A. (**A&B**) Effect of inhibiting STAT phosphorylation on B19V infection. Day 8 CD36^+^ EPCs cultured under normoxia were pre-treated with pharmacological inhibitors of phosphorylation at the indicated final concentration, or with 0.5% DMSO (control). At 24 hrs post-treatment, the cells were analyzed by Western blotting for the inhibitory effects on phosphorylation (A); or were infected with B19V (at an MOI of 5,000 gc/cell) (B). At 48 hrs, p.i., the infected cells were assessed for the level of B19V VP2-encoding mRNA per β-actin mRNA. (**C&D**) B19V infection in the context of constitutively active STAT5A and STAT5A-specific shRNAs. Day 7 CD36^+^ EPCs cultured either under normoxia (C) or hypoxia (D) were transduced with the indicated lentivirus. At 48 hrs post-transduction, the cells were infected with B19V at an MOI of 5,000 gc/cell, and at 48 hrs p.i., they were analyzed by flow cytometry for the expression of B19V NS1, STAT5A and pSTAT5A. The GFP-positive population of lentivirus-transduced cells was selectively gated. Percentages shown in the left column indicate the level of NS1-expressing cells, and the numbers in the right column the MFI of STAT5A or pSTAT5A. Results shown are representative of at least two independent experiments. Dashed reference lines in panels C&D were selected arbitrarily to show the relative positions of the peaks, and Bkg (background) represents the secondary antibody only control.

Since STAT5A was upregulated in CD36^+^ EPCs under hypoxia (Figure 6C) and inhibition of Jak2 phosphorylation is known to block B19V replication [24], we hypothesized that STAT5A signaling is critical to supporting B19V replication in CD36^+^ EPCs cultured under normoxia, and may be responsible for the increase in efficiency of B19V infection in the cells cultured under hypoxia. We tested this using three pharmacological inhibitors (the Jak2 inhibitor AG490, a STAT5B inhibitor and a STAT3 inhibitor), examining their influence on B19V infection under normoxia (Figure 7A&B). A 70% inhibition of STAT5A phosphorylation by 5 µM AG490 resulted in failure to detect the VP2-encoding mRNA (Figure 7B) in the absence of significant cytotoxicity (Figure S4A), as we had previously demonstrated [24]. At the maximum concentration of 200 µM, the STAT5B inhibitor reduced production of the VP2-encoding mRNA to ∼40% of that seen in the DMSO control. The STAT3 inhibitor had no significant effect on the level of the VP2-encoding mRNA (Figure 7B). The lesser sensitivity of B19V infection to the STAT5B inhibitor is likely due to cross-inhibition of the STAT5 SH2 domain [36] as Epo/EpoR signaling does not activate STAT5B [37]. We therefore focused on the role of STAT5A activation in B19V infection.

To confirm that STAT5A plays a critical supportive role in B19V infection of CD36^+^ EPCs, we generated two lentiviruses that express a constitutively active STAT5A, STAT5A(1*6) [38]. Expression of the constitutively active STAT5A(1*6), with the 5′ untranslated region (UTR) either present [UTRSTAT5A(1*6)] or absent [STAT5A(1*6)], in normoxia-cultured CD36^+^ EPCs led to an ∼2.5-fold increase in STAT5A phosphorylation in transduced (GFP^+^) cells over that seen in the GFP expression control (Figure 7C). As a result, the NS1-expressing cell population among the GFP^+^ cells transduced with either of the STAT5A(1*6)-expressing lentiviruses increased 2-fold compared to that in Lenti-GFP control-transduced (GFP^+^) cells (Figure 7C). These results strongly suggest that upregulation of STAT5A phosphorylation facilitates B19V infection of CD36^+^ EPCs cultured under normoxia.

From the point that STAT5A phosphorylation was increased in CD36^+^ EPCs cultured under hypoxia (Figure 6C), we generated two lentiviruses that express validated shRNAs to specifically knock down STAT5A expression in these cells. Cells treated with the shRNA-expressing lentiviruses did not show drastic change of the cell cycle and cell death (sub G0 phase) (Figure S4B), indicating that application of lentiviral vectors is safe to CD36^+^ EPCs. As shown in Figure 7D, the levels of both STAT5A expression and STAT5A phosphorylation were significantly reduced (by ∼50% and 40%, respectively), in the cells expressing the SATA5A shRNA1 and shRNA2, compared to levels in control cells expressing the scrambled shRNA (Figure 7D, panels STAT5&pSTAT5). Correspondingly, NS1 expression was significantly decreased in the cells in which STAT5A phosphorylation was reduced (Figure 7D, panel NS1).

All three lines of evidence presented here confirm that STAT5A phosphorylation is critical to B19V infection of CD36^+^ EPCs in the context of normoxia, and that elevated phosphorylation of STAT5A in CD36^+^ EPCs under hypoxia, at least partially, accounts for the enhanced B19V infection of these cells cultured under hypoxia.

### B19V infection is negatively regulated by MEK/ERK signaling

**Figure 8:**
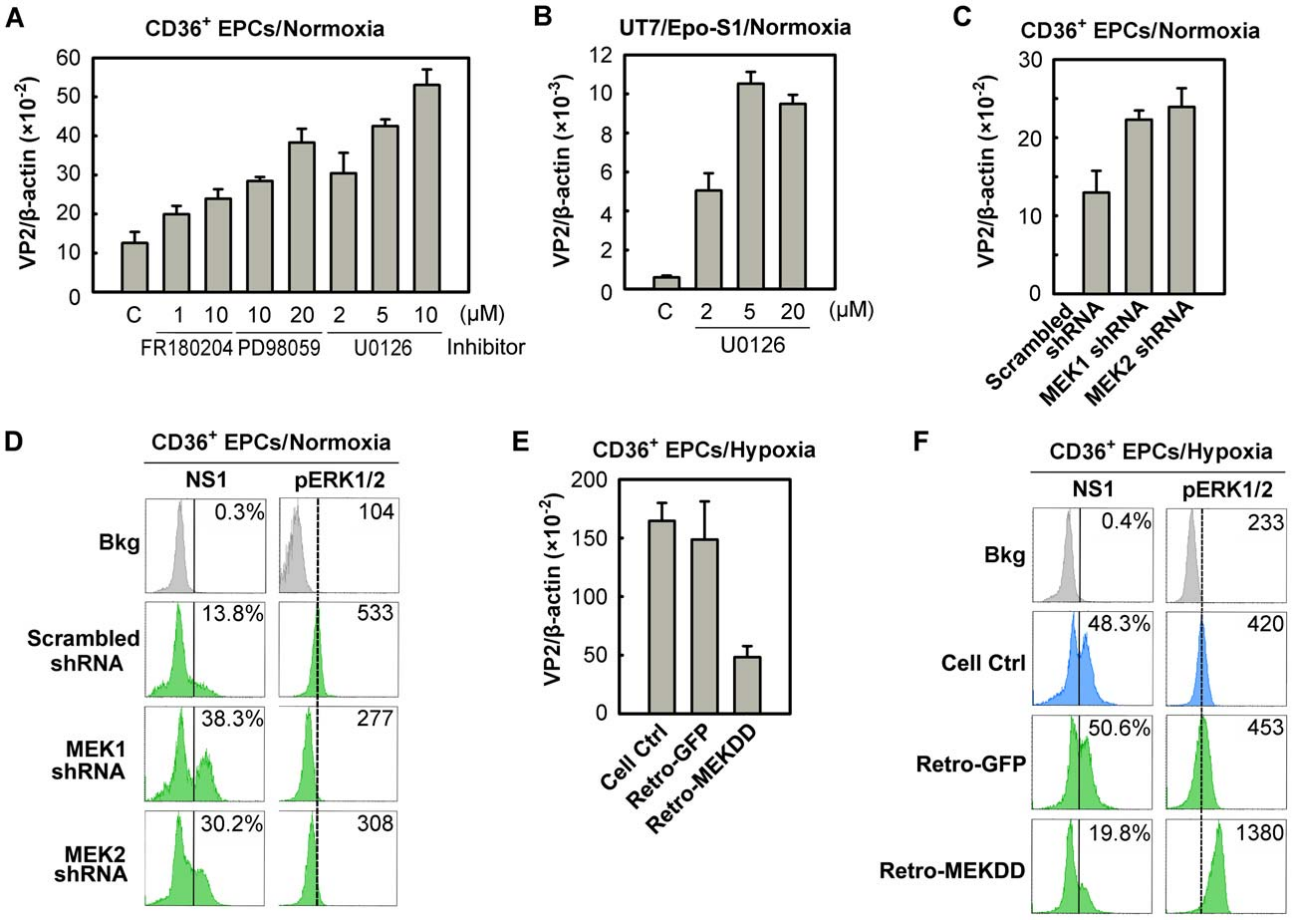
Regulation of B19V infection of CD36^+^ EPCs by MEK/ERK pathway. (**A&B**) Day 8 CD36^+^ EPCs (A) or UT7/Epo-S1 cells (B) cultured under normoxia were pre-treated with the indicated pharmacological inhibitors of MEK/ERK at 24 hrs prior to B19V infection (at an MOI of 5,000 gc/cell). At 48 hrs, p.i., the infected cells were quantified for the level of B19V VP2-encoding mRNA per β-actin mRNA. (**C&D**) Day 7 CD36^+^ EPCs cultured under normoxia were transduced with lentiviruses expressing MEK-specific shRNAs or the scrambled shRNA (control). (**E&F**) Day 7 CD36^+^ EPCs cultured under hypoxia were transduced with retroviruses expressing constitutively active MEK (Retro-MEKDD) and GFP (Retro-GFP; control). The transduced normoxia- or hypoxia-cultured CD36^+^ EPCs were infected with B19V at MOIs of 5,000 gc/cell at 48 hrs post-transduction. Groups of treated cells were quantified for the level of B19V VP2-encoding mRNA per β-actin mRNA (C&E), and analyzed for expression of B19V NS1 and phosphorylated ERK1/2 (pERK1/2) by flow cytometry. The percentages shown in the left column indicate the level of NS1-expressing cells, whereas the numbers shown in the right column represent the MFI of pERK1/2 expression (D&F). Results shown are representative of at least two independent experiments. Dashed reference lines in panels D&F were selected arbitrarily to show the relative position of the peaks, and Bkg (background) represents the secondary antibody only control.

The downregulation of ERK phosphorylation in CD36^+^ EPCs cultured under hypoxia (Figure 6C) led us to speculate that inhibition of ERK phosphorylation affects B19V infection of CD36^+^ EPCs. We thus examined the role of this pathway during B19V infection of CD36^+^ EPCs cultured under normoxia, selecting three pharmacological inhibitors of the MEK/ERK pathway: FR180204 (ERK-specific) [39], PD98059 and U0126 (MEK-specific) [40]. Application of each of these inhibitors enhanced the effectiveness of B19V infection in treated CD36^+^ EPCs, as detected by significant increases in levels of B19V VP2-encoding mRNA (Figure 8A). Most strikingly, application of 10 µM U0126, an MEK-specific inhibitor, resulted in 5-fold increase in the level of VP2-encoding mRNA. Similarly, U0126 treatment of B19V-infected UT7/Epo-S1 cells resulted in a 10-fold increase of the VP2-encoding mRNA (Figure 8B). These results suggest that inhibition of the MEK/ERK pathway promotes B19V infection in both CD36^+^ EPCs and UT7/Epo-S1 cells cultured under normoxia.

To confirm such a role for the MEK/ERK pathway during B19V infection, we generated lentiviruses expressing shRNAs validated to specifically knock down the upstream regulators MEK1 and MEK2. Inhibition of either MEK1 or MEK2 by applying MEK1 or MEK2 shRNAs led to an ∼2-fold increase in the level of the VP2-encoding mRNA (Figure 8C), a decrease in the phosphorylation of their substrate (pERK1/2), and an ∼3-fold increase in the NS1-expressing cell population (Figure 8D). Moreover, we transduced a retrovirus expressing a constitutively active MEK (MEKDD) [41] into CD36^+^ EPCs cultured under hypoxia, and tested it for a role in increasing MEK phosphorylation and interfering with B19V infection. Not surprisingly, overexpression of MEKDD resulted in elevated pERK1/2 levels (Figure 8F), a reduction in the levels of the VP2-encoding mRNA (by ∼5-fold; Figure 8E) and a reduction in the NS1-expressing cell population (∼2.5-fold; Figure 8F) compared to the corresponding levels in their GFP-expressing controls (Figure 8E&F, Retro-GFP). Together, all the results we obtained here confirm that the MEK/ERK pathway negatively regulates B19V infection of CD36^+^ EPCs cultured under both normoxia and hypoxia.

### B19V infection is not influenced by PI3K/AKT signaling

**Figure 9:**
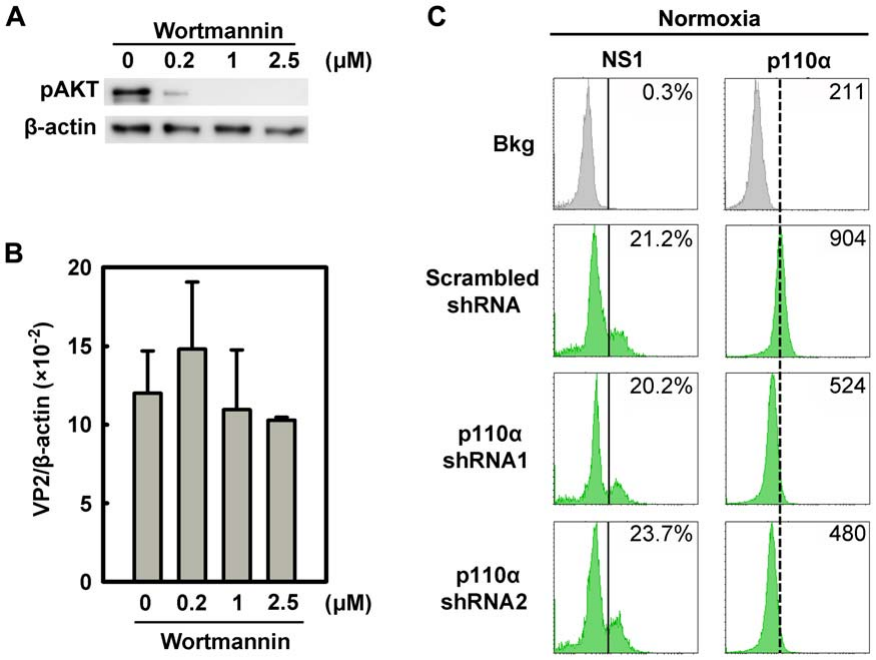
Regulation of B19V infection of CD36^+^ EPCs by the PI3K/AKT pathway. (**A&B**) Day 8 CD36^+^ EPCs cultured under hypoxia were treated with the PI3K-specifc inhibitor Wortmannin at the final concentrations indicated. At 24 hrs post-treatment, the cells were infected with B19V at an MOI of 5,000 gc/cell, and at 48 hrs p.i., were tested for AKT phosphorylation (pAKT) by Western-blotting (A). Also, the levels of VP2-encoding mRNA per β-actin mRNA were quantified (B). (**C**) Day 7 CD36^+^ EPCs cultured under normoxia were transduced with lentiviruses expressing p110α-specific shRNAs and scrambled shRNA (control), respectively. At 48 hrs post-transduction, all groups of transduced cells were infected with B19V at an MOI of 5,000 gc/cell. At 48 hrs p.i., levels of B19V NS1 and of the PI3K subunit p110α were analyzed by flow cytometry. The GFP-positive population of lentivirus-transduced cells was selectively gated. The percentages shown in the left and right columns indicate the level of NS1-expressing cells and the MFI of p110α expression, respectively. Results shown are representative of at least two independent experiments. Dashed reference lines were selected arbitrarily to show the relative position of the peaks, and Bkg (background) represents the secondary antibody only control.

To exclude a role for the PI3K/AKT pathway in B19V infection, we firstly used the PI3K-specific pharmacological inhibitor Wortmannin, to inhibit AKT phosphorylation in CD36^+^ EPCs under normoxia (Figure 9A). The application of Wortmannin at final concentrations of 0.2, 1, and 2.5 µM failed to yield a statistical difference in B19V infection, based on quantification of the VP2-encoding mRNA (Figure 9B). In addition, we employed lentiviruses that expressed shRNAs specifically targeting the p110α subunit of PI3K. Although both shRNA lentiviruses knocked down the level of p110α by ∼50%, neither affected NS1 expression in p110α shRNA-expressing (GFP^+^) cells compared with that in the cells expressing the scrambled shRNA-expressing (GFP^+^) (Figure 9C). Together with the observation that AKT was not elevated in CD36^+^ EPCs under hypoxia, these results lead us to conclude that the PI3K/AKT pathway is not directly involved in B19V infection of CD36^+^ EPCs.

### B19V infection is synergistically facilitated by expression of a constitutively active STAT5A and application of an MEK inhibitor

**Figure 10:**
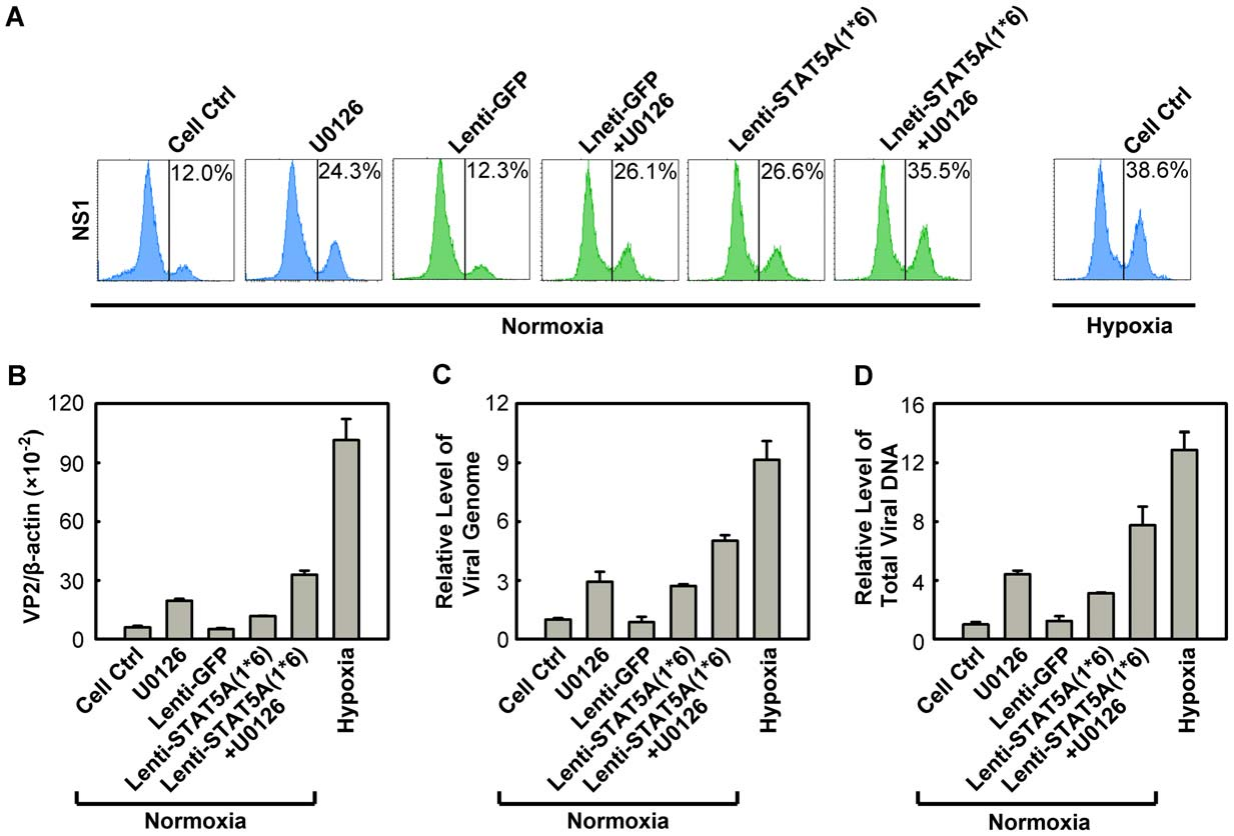
Regulation of B19V infection of CD36^+^ EPCs by combinatorial STAT5A upregulation and MEK inhibition. CD36^+^ EPCs cultured under normoxia were transduced with Lenti-STAT5A(1*6) at day 7, pre-treated with U0126 at a final concentration of 10 µM at day 8, or were subjected to both treatments. At day 9, the cells were infected with B19V (at an MOI of 4,000 gc/cell). Day 9 CD36^+^ EPCs cultured under hypoxia and infected with B19V served as a control. (**A**) At 48 hrs p.i., the GFP-positive population of lentivirus-transduced cells was selectively gated, and analyzed for NS1 expression by flow cytometry; the percentage of positive cells is indicated on right side of each panels. The GFP-positive population of lentivirus-transduced cells was selectively gated. (**B, C&D**) At 48 hrs p.i., the infected cells without selection of the GFP-positive population were quantified for the level of B19V VP2-encoding mRNA per β-actin mRNA. The numbers of virus particles (packaged viral DNA) produced (C), and the total viral DNA in the Hirt DNA samples extracted from the infected cells (D) were quantified by qPCR. The numbers shown in panels C&D are levels of these copy numbers relative to those in the control group (arbitrarily set to 1). A representative result of three independent experiments is shown in each of the panels.

Since we observed that regulation of both STAT5A and MEK/ERK pathways did not affect each other in facilitating B19V infection (Figure S5A&B), we next examined whether it is possible to further modulate B19V infection of normoxia-cultured CD36^+^ EPCs by manipulating the STAT5A and MEK/ERK pathways simultaneously. To this end, we used the constitutively active STAT5A and the U016 MEK inhibitor in combination to assess B19V infection. As shown in Figure 9A, individually U0126 treatment and STAT5A(1*6)-expression led to an increase in the NS1-expressing cell population, from ∼12% in the control groups (Figure 10A, Cell Ctrl&Lenti-GFP) to ∼24% and 26%, respectively, in transduced GFP^+^ cells. However, when the treatments were combined the NS1-expressing population was boosted to a level of 35%, a level comparable to that seen when CD36^+^ EPCs were infected under hypoxia (38%). This synergistic enhancement of B19V infection was confirmed by respective increases in the levels of the VP2-encoding mRNA, progeny virus (packaged viral genome) and total viral DNA (Figure 10B, C&D, respectively). Notably, we did not select the transduced GFP^+^ cell population [STAT5A(1*6)-expressing cells] when quantifying the levels of the B19V mRNA and DNAs, which may account for the small difference between these results and those for the hypoxia group with respect to the NS1-expressing cell percentage, which was determined in cells selected for GFP expression. The lentivirus transduction efficiency (GFP^+^ rate) was ∼50%.

These results demonstrated that we have recapitulated the increased B19V infection of CD36^+^ EPCs under hypoxia by manipulating phosphorylation of both STAT5A and ERK in CD36^+^ EPCs under normoxia.

## Discussion

B19V infection shows a remarkable tropism for human erythroid progenitors in the bone marrow of infected individuals [42], [43]. The microenvironment of bone marrow in both animals and humans has been suggested to be hypoxic relative to other tissues, and both HSCs and EPCs are thought to reside in the most hypoxic microenvironment within the bone marrow [44]–[46]. In the current study, we have mimicked the natural “niches” of EPCs in the bone marrow, demonstrating that B19V infection of *ex vivo* expanded EPCs cultured under hypoxia is conducive to sustained production of progeny B19V *in vitro*. Our results show that even in the absence of changes in virus entry or intracellular trafficking, the hypoxic environment leads to fine tuning of nuclear microenvironment of B19V-infected EPCs, by regulating phosphorylation of STAT5A and MEK, and thereby boosting the production of B19V progeny virus over 10-fold.

### Hypoxia enhances replication of the B19V genome in the nucleus

The enhancement of B19V infection in EPCs cultured under hypoxia is due to the increased replication of the viral genome after it enters the nucleus of cells in the context of hypoxia (Figure 3A–D). More direct evidence that transfected B19V infectious DNA replicated ∼80 times more rapidly in UT7/Epo-S1 cells under hypoxia than under normoxia further supports the notion that hypoxia facilitates replication of the viral genome in the nucleus. Our study is the first to show that the ssDNA B19V genome and an a high level, up to ∼150 ffu/µl, of progeny virus are produced following transfection of the B19V infectious DNA into UT7/Epo-S1 cells [26]. However, the ratio of ssDNA/RF DNA (1∶4) remained lower than that observed during B19V infection of EPCs (∼1∶1). This is likely due to the semi-permissiveness of UT7/Epo-S1 cells to B19V infection; even following infection, progeny virus is produced inefficiently [13], [47]. Given that parvovirus DNA replication occurs by a “rolling hairpin” model [4], i.e., synthesis of the ssDNA genome and its packaging into the assembled virion take place simultaneously [48]. We believe that replication of the B19V RF DNA, but not generation of the ssDNA B19V genome or capsid assembly, is the key event of the B19V life cycle that is elevated in B19V-infected EPCs under hypoxia. Thus, our study has provided a novel tool for performing reverse genetics of B19V, which has not been possible before this study [26], [49].

### In the context of hypoxia, the canonical PHD/VHL/HIFα pathway does not contribute to the regulation of B19V infection

In the context of normoxia, HIFα is hydroxylated by PHDs and thus targeted by an E3 ubiquitin ligase in the VHL (von Hippel-Lindau protein) complex, followed through a proteasome-mediated degradation pathway [19]. In the context of hypoxia, by contrast, the activities of PHD1-3 are downregulated and the increased HIFα activity both promotes the expression of genes whose products sense hypoxia and activates signal transduction pathways that lead to physiologically appropriate changes [29], [30]. In our studies, under hypoxia, HIFα knockdown failed to affect B19V infection (Figure 5E&S2); and in the context of normoxia, although PHD1-3 knockdown resulted in a significant increase in the level of HIF1α even in the context of normoxia, it failed to affect B19V infection (Figure 5F). Moreover, regardless of whether HIF1α expression in EPCs was increased under normoxia (using shRNAs to specially knock down PHD1-3) or decreased under hypoxia (using shRNAs to specially knock down HIF1α), the phosphorylation of both STAT5A and ERK was not affected (Figure S6). Therefore, our results indicate that hypoxia-enhanced B19V infection of EPCs is independent of both HIFα and PHD. Strikingly, only a few cases of hypoxia-responding stress that are independent of the canonical PHD/VHL/HIFα pathway have been reported [19], [50]. Therefore, our studies on the hypoxia-enhanced B19V infection of EPCs demonstrate the involvement of two novel mechanisms (i.e., hypoxia-induced STAT5A and hypoxia-suppressed MEK signaling) in the regulation of hypoxia-responding EPC stress, independent of HIFα/PHD, and suggesting that these create a “niche” for B19V DNA replication in the nucleus.

### Hypoxia-cultured EPCs possess less differentiation potential than their normoxia-cultured counterparts

EPCs expanded under either condition are at similar stages of differentiation phenotypically (Figure 1A). Notably, however, probing of the intracellular markers of erythroid differentiation (GATA1, GATA2 and hemoglobin-γ) revealed that hypoxia-cultured EPCs are slightly less differentiated than their normoxia-cultured counterparts (Figure 6C). This is consistent with a recent report that expression of erythroid transcription factors, e.g., GATA1 and EKLF, was delayed and decreased in EPCs cultured under hypoxia (at 2% O_2_) [51]. GATA1 and GATA2 are erythroid lineage-specific transcription factors that specifically bind to and activate genes important for the proper differentiation of erythroid cells [52]. In this context, the decrease in GATA1 usually results in a low expression level of EpoR. However, in the case of EPCs cultured under hypoxia, more EpoR was expressed at the cell surface in spite of the fact that the levels of phosphorylated EpoR were low (Figure 6A). This finding supports the notion that negative feedback inhibits over-activation of EpoR [53]. Upon Epo ligation, EpoR is immediately phosphorylated, and this leads to a rapid receptor internalization and degradation, by both proteasomal and lysosomal mechanisms [54]. We speculate that in EPCs under hypoxia, either EpoR phosphorylation is less sensitive to Epo ligation, or the rate of ligation is slower than that in the cells under normoxia. This may leads to slower EpoR internalization and degradation, manifesting phenotypically as longer retention on the cell surface, and reduced phosphorylated EpoR intracellularly. The reduced level of phosphorylated EpoR leads to downregulation of GATA1 and GATA2 in EPCs cultured under hypoxia. Thus, we suggest that EPCs are less differentiated in the context of hypoxia, and that the apparent increase in EpoR in these cells is likely the result of decreased degradation.

### STAT5A positively regulates B19V infection

In cells of the erythroid lineage, STAT5A is generally considered to be phosphorylated by Jak2 [25]. Several important STAT5A target genes, such as Oncostatin M, Pim, SOCS, Bcl-xL and D-type cyclins, are required for erythropoiesis [25]. However, Jak2-independent STAT5A phosphorylation has also been reported in cells of erythroid lineage [55]. This is further supported by our results that although hypoxia did not lead to changes in levels of phosphorylated Jak2, STAT5A phosphorylation was increased over 2-fold (Figure 6C). We thus hypothesize that in EPCs STAT5A may be a substrate for other kinases in addition to Jak2, at least in the context of hypoxia. STAT5A-driven erythroid differentiation is largely dependent on the erythroid-specific transcription factor GATA1, and STAT5A-driven proliferation appears to be independent of GATA1 [56]. However, over-activation of STAT5A does not induce GATA1 expression significantly [57]. In fact, we observed that GATA1 levels were decreased in EPCs cultured under hypoxia whereas STAT5A was upregulated, suggesting that STAT5A-actviated B19V DNA replication is likely GATA1-independent. We hypothesize that these GATA1-independent STAT5-targeted genes [56], e.g., Oncostatin M, Pim and SOCS, likely play critical roles in regulating B19V DNA replication.

### The MEK/ERK pathway negatively regulates B19V infection

The MEK/ERK pathway is critical to Epo stimulation-dependent erythroid cell proliferation and survival, which is mediated by the Grb2-Ras-Raf1 pathway [25]. The levels of ERK expression and activation fine-tune the balance between proliferation and differentiation of erythroid progenitors [58]. In EPCs under hypoxia, the decrease in EpoR phosphorylation may result in a reduction of ERK phosphorylation (Figure 6C), and this explains the reduced EPC proliferation in the context of hypoxia (Figure S3A&B). Despite the slight change in differentiation status and slow proliferation rate of EPCs under hypoxia, these cells still expressed similar levels of the major erythroid phenotypic markers and B19V receptors, suggesting that the balance between the two processes keeps them moving through erythropoiesis.

The MEK/ERK pathway has been shown to be upregulated during infection by various viruses, and in the context of certain RNA and DNA viruses has been implicated as a positive regulator of both virus entry and intracellular trafficking during infection [59], for some DNA viruses as a positive regulator of virus replication through regulating the cell cycle [60]. Notably, inhibition of MEK or its substrate ERK significantly decreases virus infection. Our findings clearly show that the MEK/ERK pathway is a negative regulator of B19V infection in EPCs, a role unique among those for this pathway in viral infection. Notably, the B19V small non-structural 11 kDa protein has been shown *in vitro* to interact with Grb2 specifically [61], a crucial adaptor for the activation of Ras/Raf1 and, in turn, for MEK/ERK signaling, which is activated by phosphorylation of EpoR tyrosine residue 489 [25]. These lines of evidence lead us to postulate that, during B19V infection, 11 kDa interacts with Grb2 to inhibit MEK/ERK signaling, thereby facilitating B19V DNA replication.

In erythroid cells, the ERK1/2 pathway is involved in the early proliferative phases of erythropoiesis [34], and in the inhibition of terminal erythroid differentiation [33]. Our discovery that S-phase was delayed in hypoxia-cultured EPCs (Figure S4B), and that hypoxia inhibited cell proliferation ∼2-fold, can be explained by decreased level of ERK under hypoxia. Notably, B19V infection of EPCs showed a remarkable inhibition of cell proliferation, and cell cycle arrest [62]. Although this B19V-induced anti-proliferation effect and cell cycle arrest have been shown to be beneficial to B19V replication, the underlying mechanism remains unknown [62]. ERK1/2 translocate to the nucleus, and directly or indirectly phosphorylate many transcription factors [63]. We hypothesize that the reduced ERK1/2 activation in EPCs produces an optimal microenvironment for B19V DNA replication in the nucleus. Given that downregulation of the MEK/ERK pathway does not increase phosphorylation of STAT5A (Figure S5), the two pathways appear to function independently.

In conclusion, the balanced homeostasis of EPCs under hypoxia, accompanied by the upregulation of phosphorylated STAT5A and downregulation of ERK activity, provides B19V with a nuclear microenvironment optimal for replication of its genome, independent of HIFα expression. Thus, our study reveals the factors that are critical to B19V replication and raise infection of EPCs to a productive level, in a process that likely mimics native B19V infection of human bone marrow.

## Materials and Methods

### CD36^+^ EPCs and UT7/Epo-S1 cells

#### *Ex vivo* expansion of CD36^+^ EPCs

CD34^+^ hematopoietic stem cells (HSCs) derived from human bone marrow were purchased from the National Disease Research Interchange (NDRI), Philadelphia, PA. Upon arrival in the laboratory (defined as day 0), these cells were cultured in CD36^+^ EPC expansion medium [16], [27] at 5% CO_2_ and 21% O_2_ at 37°C (normoxia) until day 4, and were then frozen in liquid nitrogen at 0.5×10^6^ cells/vial. These cells were defined as day 4 HSCs [16], [27]. The Day 4 HSCs were thawed and cultured in the same expansion medium, under either normoxia (5% CO_2_ and 21% O_2_) or hypoxia (5% CO_2_ and 1% O_2_) at 37°C until the times of treatment and B19V infection as indicated in the figure legends. The hypoxia condition was achieved using the three-door chamber of the HERAcell 150 tri-gas incubator (Thermo Fisher).

#### UT7/Epo-S1 cells

UT7/Epo-S1 cells were obtained from Dr. Kevin Brown, with permission from Dr. Kazuo Sugamura, and were grown as described previously [11], [47].

### Virus and virus infection

Viremic plasma (no.: P32) was obtained from ViraCor-IBT Laboratories (Lee's Summit, MO), and the numbers of B19V genome copies (gc) per milliliter (10^12^ gc/ml) was quantified as previously described [64]. B19V infection was carried out by adding the B19V-containing plasma or lysates of infected cells directly to the culture. Multiplicity of infection (MOI) used for each experiment is indicated in the corresponding figure legend.

### Nucleofection

UT7/Epo-S1 cells were electroporated with 2 µg of a B19V infectious DNA (M20), which was digested from the B19V infectious clone pM20 [26], or indicated plasmids using the Amaxa Nucleofector (Lonza) as described previously [27].

### Assessment of virus infection

#### Immunofluorescence assay

Immunofluorescence assay was performed followed a protocol described previously [47]. First antibodies were as follows: rat anti-B19V NS1, anti-11 kDa antiserum [27], mouse anti-capsid monoclonal antibody (Millipore, MAB8293) at 1∶100 dilution in PBS-FCS, for 1 hrs at 37°C. Secondary antibodies were fluorescein isothiocyanate (FITC)-conjugated anti-rat IgG second antibody (Jackson ImmunoResearch Laboratories) and FITC-conjugated anti-mouse IgG (F9006, Sigma). DAPI was used to stain the nucleus.

#### Reverse transcription (RT)–quantitative PCR (qPCR) for quantification of viral mRNA transcripts, qPCR for quantification of replicated viral DNA and packaged viral genomes (virus particles) and virus entry assay

All these assays were performed as described previously [24].

#### Quantification of virus infectious units

The infectious units in B19V-containing cell lysates were titrated as fluorescence focus-forming units (ffu) per µl following the same protocol described previously [13].

#### Southern blot analysis of viral DNAs

Hirt DNA was extracted from the cells and analyzed using a B19V probe as described previously [64]. Blots were exposed to a GE phosphor imaging screen, and quantified using a Phosphor imager (Typhoon 9400) and the Image Quant TL software v2005 (GE Healthcare).

### Virus trafficking assay

Either normoxia- or hypoxia-cultured CD36^+^ EPCs were infected with B19V on day 8 of culture at an MOI of 5,000 gc/cell. Cells were harvested at the indicated time points (hrs p.i.). At 2 hrs p.i., cell-surface bound virions were removed by treatment with trypsin (0.25% trypsin in 20 mM EDTA) for 5 min at 37°C with manual agitation. The cells were washed with PBS, and nuclear fraction was prepared using the Nuclei EZ Prep Nuclei isolation Kit (NUC-101, Sigma) and following the manufacturer's instructions. The fractions were stored at −80°C until analysis. The numbers of viral genomes in the nuclear fraction were quantified by qPCR as described above, and were divided by the number of the cells collected.

### Construction, production, and transduction of retroviral and lentiviral vectors

#### Lentiviral vectors expressing shRNAs

Plasmids pLKO-GFP and pLKO-GFP-Scramble-shRNA were described previously [24]. The validated shRNA sequences shown in Table S1 were cloned into pLKO-GFP using the AgeI and EcoRI sites, to generate the pLKO-GFP-XXX-shRNA plasmids.

#### Lentiviral vectors expressing proteins

Plasmid pLenti-GFP-Puro (pLenti) was obtained from Addgene Inc. (Cambridge, MA). The GFP coding region was replaced with IRES-GFP (Clontech) using the BamHI and BsrGI sites, and multiple restriction enzyme sites (EcoRI-EcoRV-SpeI) were added between the BamHI and IRES sequences, to produce pLenti-MCS-IRES-GFP. The coding sequences of two constitutively active forms of STAT5A [UTR STAT5A(1*6) (nt 242–2715) and STAT5A(1*6) (nt 334–2715)] were amplified from pMX-STAT5A(1*6)-IRES-GFP (H298R/S710F) [65], and were inserted between the BamHI and SpeI sites of pLenti-MCS-IRES-GFP, generating pLenti-UTR-STAT5A(1*6)-IRES-GFP and pLenti-STAT5A(1*6)-IRES-GFP, respectively.

Plasmid pLenti-P6-GFP was constructed by replacing the CMV promoter sequence of the pLenti vector between the ClaI and XbaI sites with the B19V sequence spanning nt 187–564 (GenBank accession no.: AY386330). The HBS mutant was generated by mutating the putative HBS motif from 5′ACGT3′ (nt 204–207) to 5′TTTT3′ [18].

#### Retroviral vector expressing constitutively active MEK (MEKDD)

The constitutively active MEK gene, MEKDD(S218D&S222D) [41], which was amplified from pBabe-Puro-MEK-DD (Addgene), was inserted into the BamHI-XbaI-digested pMSCV-MCS-IRES-GFP-WRE vector [24], to produce pMSCV-MEKDD-IRES-GFP.

#### Production and concentration of viral vectors

Lentivirus was produced and concentrated according to instructions from Addgene (http://www.addgene.org/plko).

The retroviruses Retro-MEKDD and Retro-GFP (control) were produced by transfecting GP293 cells (Clontech) with pMSCV-MEKDD-IRES-GFP and pMSCV-MSC-IRES-GFP-WRE [24], respectively, together with pCMV-VSVG. The retroviral vectors were concentrated following the manufacturer's instructions (Clontech, cat. No.:PT3132-1).

#### Lentiviral and retroviral transduction

Concentrated lentiviral or retroviral vector was added to CD36^+^ EPCs at day 7 of culture, at an MOI of ∼4 ffu/cell.

### Pharmacological inhibitors used in this study

Wortmannin (681675), R59949 (266788), AG490 (658401), a STAT5B inhibitor (573108), a STAT3 Inhibitor (573102), FR180204 (328007), PD98059 (513000) and U0126 (662005) were purchased from EMD Chemicals, and dissolved in DMSO to generate the recommended stock solutions.

### Western blot analysis

Western blot analysis was carried out as previously described [6]. Antibodies used for Western blotting were as follows: anti-HIF1α (610959) from BD Biosciences; anti-GATA1 (sc-1234), anti-pGATA1 (sc-101687), anti-GATA2 (sc-9008), and anti-hemoglobin-γ (sc-21756) from Santa Cruz; anti-EpoR (ab56310) and anti-pSTAT5B (ab52211) from Abcam; anti-pSTAT5A (A00253), anti-pJak2 (A00360) and pSTAT3 (A00251) from GenScript; anti-pERK (4377S) and pAKT (4060S) from Cell Signaling; anti-pEpoR (2585-1) from Epitomics; and anti-β-actin (A5441) from Sigma. Secondary antibodies were HRP-conjugated anti-mouse (A4416) or HRP-conjugated anti-rabbit (A0545) from Sigma. β-actin was used as a loading control.

### Flow cytometry analysis

Cell surface staining was performed essentially as described previously [24]. The following antibodies were used: Anti-CD34 (340862), CD41 (555465), CD36 (555453), CD71 (554889), CD235a (555569), and CD49e (555615), purchased from BD Biosciences; anti-KU80 (NA52) from CalBiochem; anti-Globoside (1960) from Matreya; and anti-EpoR (ab56310) from Abcam.

Intracellular staining was performed at room temperature, essentially as described previously [66]. The following antibodies were used: HIF1α (610959) and p110α (611398), purchased from BD Biosciences; anti-pJak2 (A00360), anti-STAT5A (A00280) and anti-pSTAT5A (A00253) from GenScript; anti-pERK (4377) from Cell Signaling; and anti-pEpoR (c-20236-R) from Santa Cruz. For flow cytometry by GFP selection, the secondary antibody used was Cy5-conjugated to one of the following: anti-mouse (115-176-146), anti-rat (112-176-143) or anti-rabbit (111-176-144) from Jackson ImmunoResearch. In all other analyses, the secondary antibody was: FITC-conjugated anti-mouse IgG (715-095-151) from Jackson ImmunoResearch; anti-mouse IgM (F9529); or anti-rabbit IgG (F9887) from Sigma.

## Supporting Information

Figure S1 **In UT7/Epo-S1 cells, hypoxia also significantly increases B19V infection, and HIF1α does not activate the B19V P6 promoter.** (**A&B**) UT7/Epo-S1 cells were infected with B19V at an MOI of 20,000 gc/cell under either normoxia (N) or hypoxia (H). At 48 hrs p.i., copy numbers of total viral DNA in infected cells were quantified by qPCR; the levels of B19V VP2-encoding mRNA per β-actin mRNA in infected cells were quantified. (**B**) HIF1α was detected in UT7/Epo-S1 cells cultured under normoxia (N) or hypoxia (H) by Western blotting. (**C, D&E**) UT7/Epo-S1 cells cultured under normoxia were treated with CoCl_2_ at the final concentrations indicated. CoCl_2_ (CX1800) was purchased from EMD Biochemicals, and dissolved in distilled H_2_O. At 24 hrs post-treatment, cells were detected for HIF1α stabilization by Western blotting (C). The cells also were transfected with a GFP reporter plasmid (pcDNA-P6-GFP) (diagramed in panel D). At 48 hrs post-transfection, GFP expression in the cells of each group was quantified as MFI using flow cytometry. The MFI in the “0” group is arbitrarily set up as 1, and relative MFI is shown (E). (**F**) CoCl_2_ did not show any cytotoxicity at a final concentration of 200 µM. UT7/Epo-S1 cells were cultured under normoxia and treated with CoCl_2_ at indicated concentrations. Cytotoxicity was evaluated by the CellTiter-Glo® kit at 48 hrs post-treatment.
(TIF)

Figure S2 **HIF2α and HIF3α do not affect B19V infection of CD36^**+**^ EPCs cultured under hypoxia.** Day 7 CD36^+^ EPCs cultured under hypoxia were transduced with the indicated lentivirus at 48 hrs prior to B19V infection (at an MOI of 2,000 gc/cell). At 48 hrs p.i., transduced cells were analyzed for expression of B19V NS1, HIF2α and HIF3α by flow cytometry. Percentages of NS1-positve cells are shown in the first column, and numbers shown in the middle and right columns are MFIs of HIF2α and HIF3α, respectively. Dashed reference lines are drawn arbitrarily to show the relative position of the peaks. Bkg, background, represents the second antibody control.
(TIF)

Figure S3 **Proliferation, viability and cell cycle analysis of CD36^**+**^ EPCs cultured under normoxia** ***vs.*** **hypoxia.** (**A**) The total numbers of CD36^+^ EPCs cultured under either normoxia or hypoxia were counted on each day of culture using hemocytometer and plotted to the days of culture. (**B**) Proliferation of CD36^+^ EPCs cultured under either normoxia or hypoxia was determined using the CellTiter-Glo® kit for intracellular ATP. The value on day 4 is arbitrarily set up as 1, to which the relative values are plotted to the days of culture. (**C**) The cell cycle was analyzed by DAPI staining, and the percentages of each cell cycle phase in the live cell population are shown on the top right corner in each plot. The sub G0 population, as shown vertically to the left of each panel, represents levels of cell death.
(TIF)

Figure S4 **Cytotoxicity detection of the pharmacological inhibitors and lentiviral vectors used in the study.** (**A**) CellTiter-Glo® kit was used to determine viability of the cells at 48 hrs post-treatment with chemicals indicated. The final concentrations of each inhibitor tested are shown. The highest concentration of DMSO used to dissolve the inhibitors was 0.5%, which was therefore used as a DMSO control. The value determined from the cell only control group is arbitrarily set up as 1, and relative viability is shown. (**B**) Day 7 CD36^+^ EPCs cultured under normoxia and hypoxia indicated were transduced using respective shRNA-encoding lentivirus as shown. Flow cytometry analysis was carried out at 48 hrs post-transduction to determine the cell cycle status in each group. The percentage of sub G0 was presented as an indicator of cell death. The percentage of cells at G0/G1, S or G2/M phase was the relative value of non-Sub G0 cells.
(TIF)

Figure S5 **Phosphorylation of STAT5A and MEK does not affect each other significantly.** (**A**) Day 7 CD36^+^ EPCs cultured under normoxia were either transduced with MEK-targeted shRNA (MEK shRNA) and scrambled shRNA, respectively, or treated with MEK-specific inhibitor U0126 at a final concentration of 10 µM. (**B**) Day 7 CD36^+^ EPCs cultured under normoxia were transduced with Lenti-GFP and Lenti-UTRSTAT5A(1*6). At 48 hrs post-transduction or post-treatment, the levels of pSTAT5A (A) and pERK1/2 (B) were analyzed by flow cytometry. The GFP-positive population of lentivirus-transduced cells was selectively gated. Numbers shown are MFI of the whole peak. Dashed reference line is selected arbitrarily to show the relative position of the peaks. Bkg, background, secondary antibody only.
(TIF)

Figure S6 **HIFα does not modulate phosphorylation of STAT5A and MEK1/2 under normoxia and hypoxia.** Day 7 CD36^+^ EPCs cultured under either normoxia or hypoxia were transduced by respective shRNA-encoding lentivirus. The levels of phosphorylated ERK1/2 (pERK1/2) and phosphorylated STAT5A (pSTAT5A) were determined by flow cytometry at 48 hrs post-transduction. The dash line was drawn arbitrarily to show the shift of staining peak from the background control (Bkg). The numbers in the plots of the left and right columns show MFI values of pERK1/2 and pSTAT5A, respectively.
(TIF)

Table S1 **Sequences of shRNAs used in this study.**
(DOC)

Text S1 **Supplemental methods.**
(DOC)
