# Supplementary figures and images for: Productive Parvovirus B19 Infection of Primary Human Erythroid Progenitor Cells at Hypoxia Is Regulated by STAT5A and MEK Signaling but not HIFα
Source: PLoS Pathog. 2011 Jun 16;7(6):e1002088. doi: 10.1371/journal.ppat.1002088 (PMC3116823; doi:10.1371/journal.ppat.1002088)

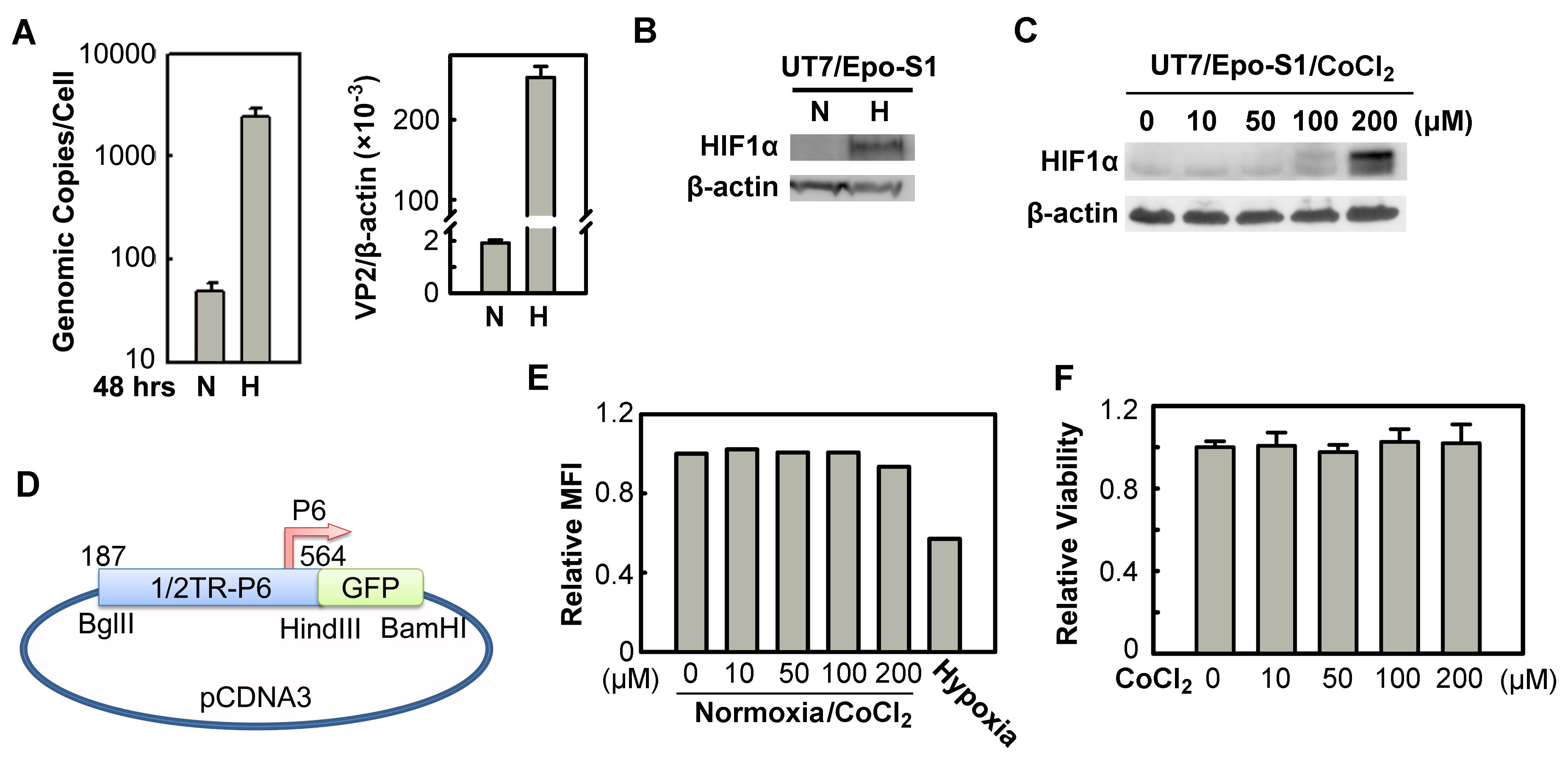

Supplement: Figure S1 — In UT7/Epo-S1 cells, hypoxia also significantly increases B19V infection, and HIF1α does not activate the B19V P6 promoter. (A&B) UT7/Epo-S1 cells were infected with B19V at an MOI of 20,000 gc/cell under either normoxia (N) or hypoxia (H). At 48 hrs p.i., copy numbers of total viral DNA in infected cells were quantified by qPCR; the levels of B19V VP2-encoding mRNA per β-actin mRNA in infected cells were quantified. (B) HIF1α was detected in UT7/Epo-S1 cells cultured under normoxia (N) or hypoxia (H) by Western blotting. (C, D&E) UT7/Epo-S1 cells cultured under normoxia were treated with CoCl2 at the final concentrations indicated. CoCl2 (CX1800) was purchased from EMD Biochemicals, and dissolved in distilled H2O. At 24 hrs post-treatment, cells were detected for HIF1α stabilization by Western blotting (C). The cells also were transfected with a GFP reporter plasmid (pcDNA-P6-GFP) (diagramed in panel D). At 48 hrs post-transfection, GFP expression in the cells of each group was quantified as MFI using flow cytometry. The MFI in the “0” group is arbitrarily set up as 1, and relative MFI is shown (E). (F) CoCl2 did not show any cytotoxicity at a final concentration of 200 µM. UT7/Epo-S1 cells were cultured under normoxia and treated with CoCl2 at indicated concentrations. Cytotoxicity was evaluated by the CellTiter-Glo® kit at 48 hrs post-treatment. (TIF) [file ppat.1002088.s001.tif]

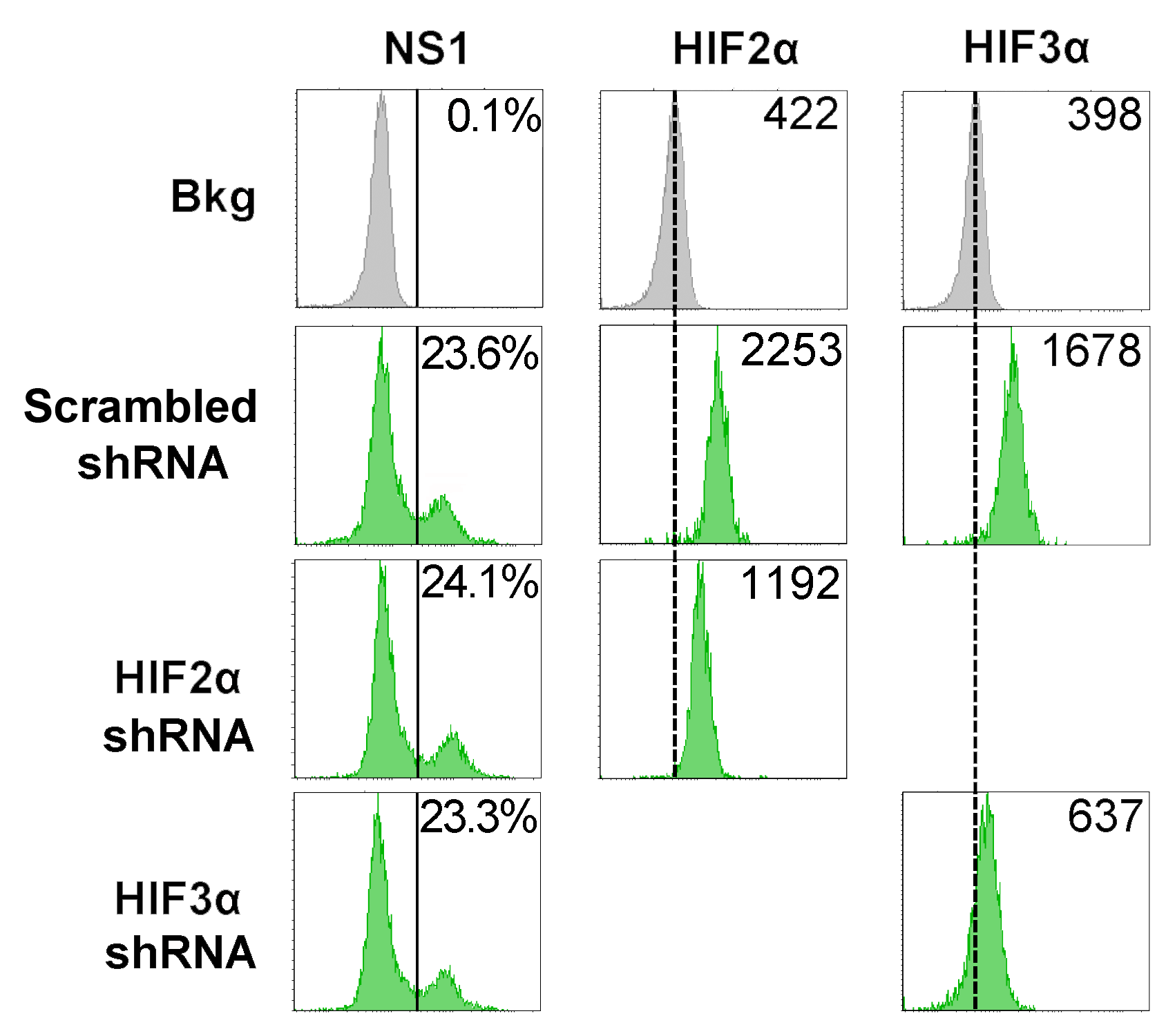

Supplement: Figure S2 — HIF2α and HIF3α do not affect B19V infection of CD36+ EPCs cultured under hypoxia. Day 7 CD36+ EPCs cultured under hypoxia were transduced with the indicated lentivirus at 48 hrs prior to B19V infection (at an MOI of 2,000 gc/cell). At 48 hrs p.i., transduced cells were analyzed for expression of B19V NS1, HIF2α and HIF3α by flow cytometry. Percentages of NS1-positve cells are shown in the first column, and numbers shown in the middle and right columns are MFIs of HIF2α and HIF3α, respectively. Dashed reference lines are drawn arbitrarily to show the relative position of the peaks. Bkg, background, represents the second antibody control. (TIF) [file ppat.1002088.s002.tif]

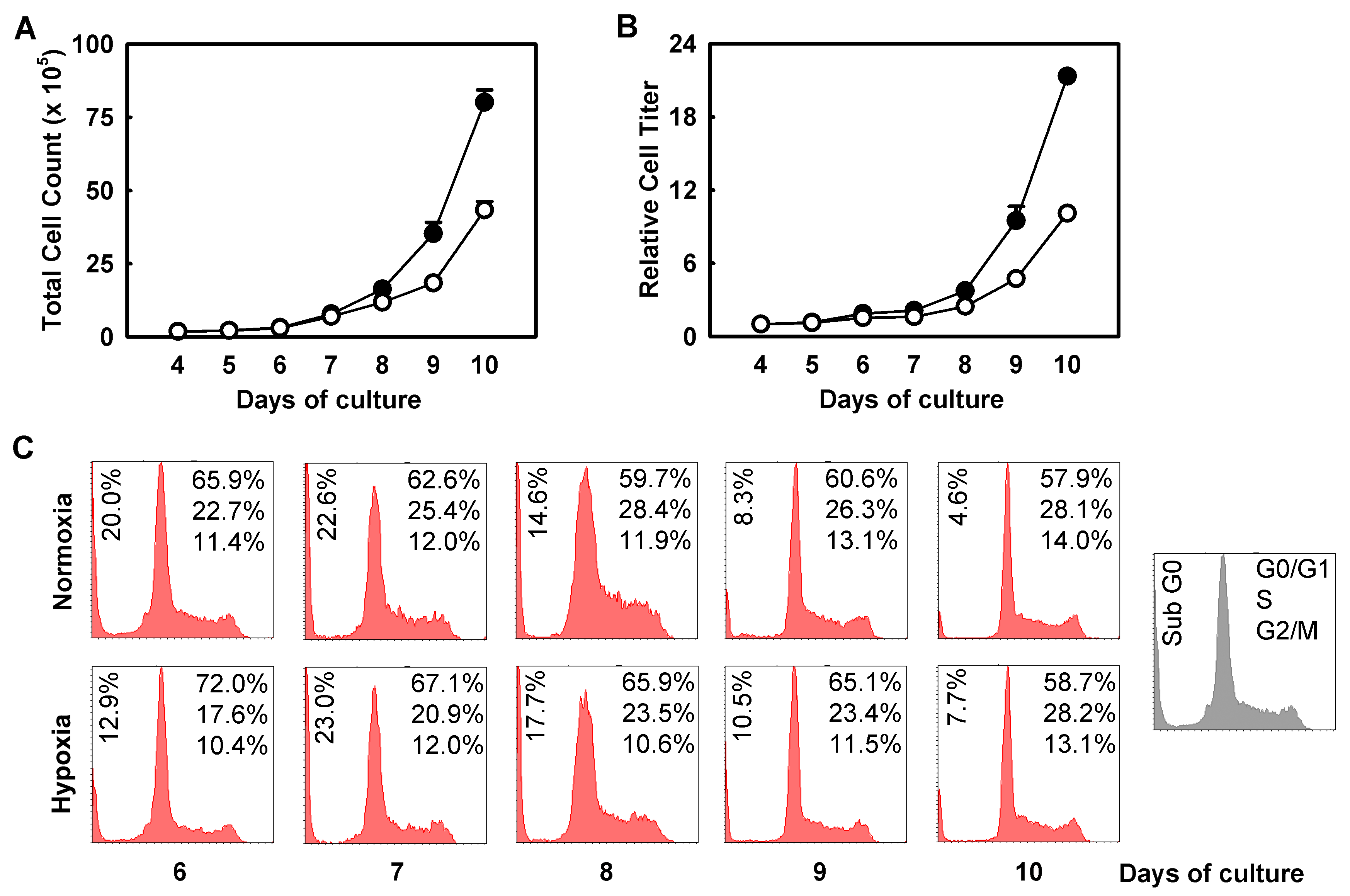

Supplement: Figure S3 — Proliferation, viability and cell cycle analysis of CD36+ EPCs cultured under normoxia vs. hypoxia. (A) The total numbers of CD36+ EPCs cultured under either normoxia or hypoxia were counted on each day of culture using hemocytometer and plotted to the days of culture. (B) Proliferation of CD36+ EPCs cultured under either normoxia or hypoxia was determined using the CellTiter-Glo® kit for intracellular ATP. The value on day 4 is arbitrarily set up as 1, to which the relative values are plotted to the days of culture. (C) The cell cycle was analyzed by DAPI staining, and the percentages of each cell cycle phase in the live cell population are shown on the top right corner in each plot. The sub G0 population, as shown vertically to the left of each panel, represents levels of cell death. (TIF) [file ppat.1002088.s003.tif]

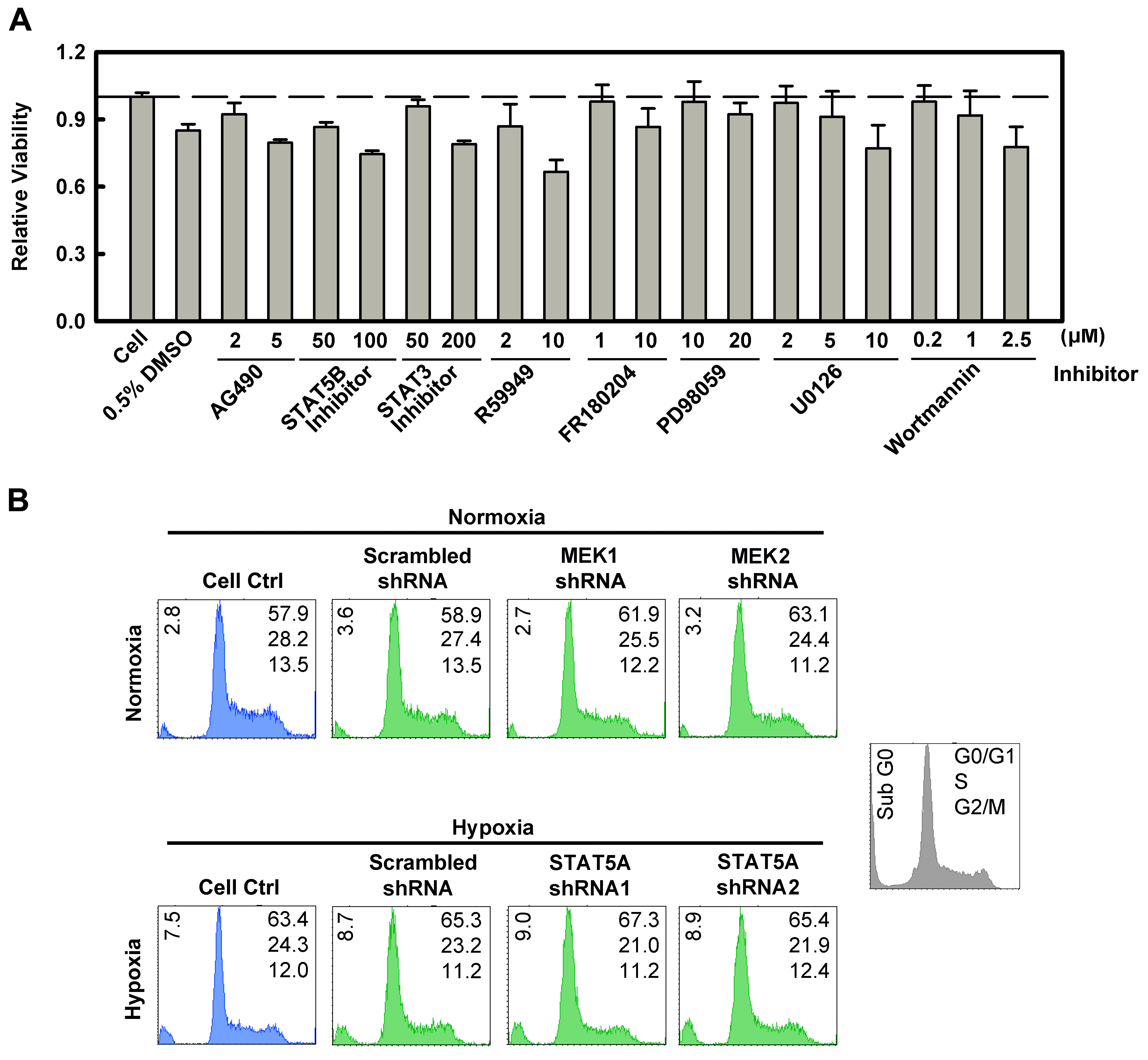

Supplement: Figure S4 — Cytotoxicity detection of the pharmacological inhibitors and lentiviral vectors used in the study. (A) CellTiter-Glo® kit was used to determine viability of the cells at 48 hrs post-treatment with chemicals indicated. The final concentrations of each inhibitor tested are shown. The highest concentration of DMSO used to dissolve the inhibitors was 0.5%, which was therefore used as a DMSO control. The value determined from the cell only control group is arbitrarily set up as 1, and relative viability is shown. (B) Day 7 CD36+ EPCs cultured under normoxia and hypoxia indicated were transduced using respective shRNA-encoding lentivirus as shown. Flow cytometry analysis was carried out at 48 hrs post-transduction to determine the cell cycle status in each group. The percentage of sub G0 was presented as an indicator of cell death. The percentage of cells at G0/G1, S or G2/M phase was the relative value of non-Sub G0 cells. (TIF) [file ppat.1002088.s004.tif]

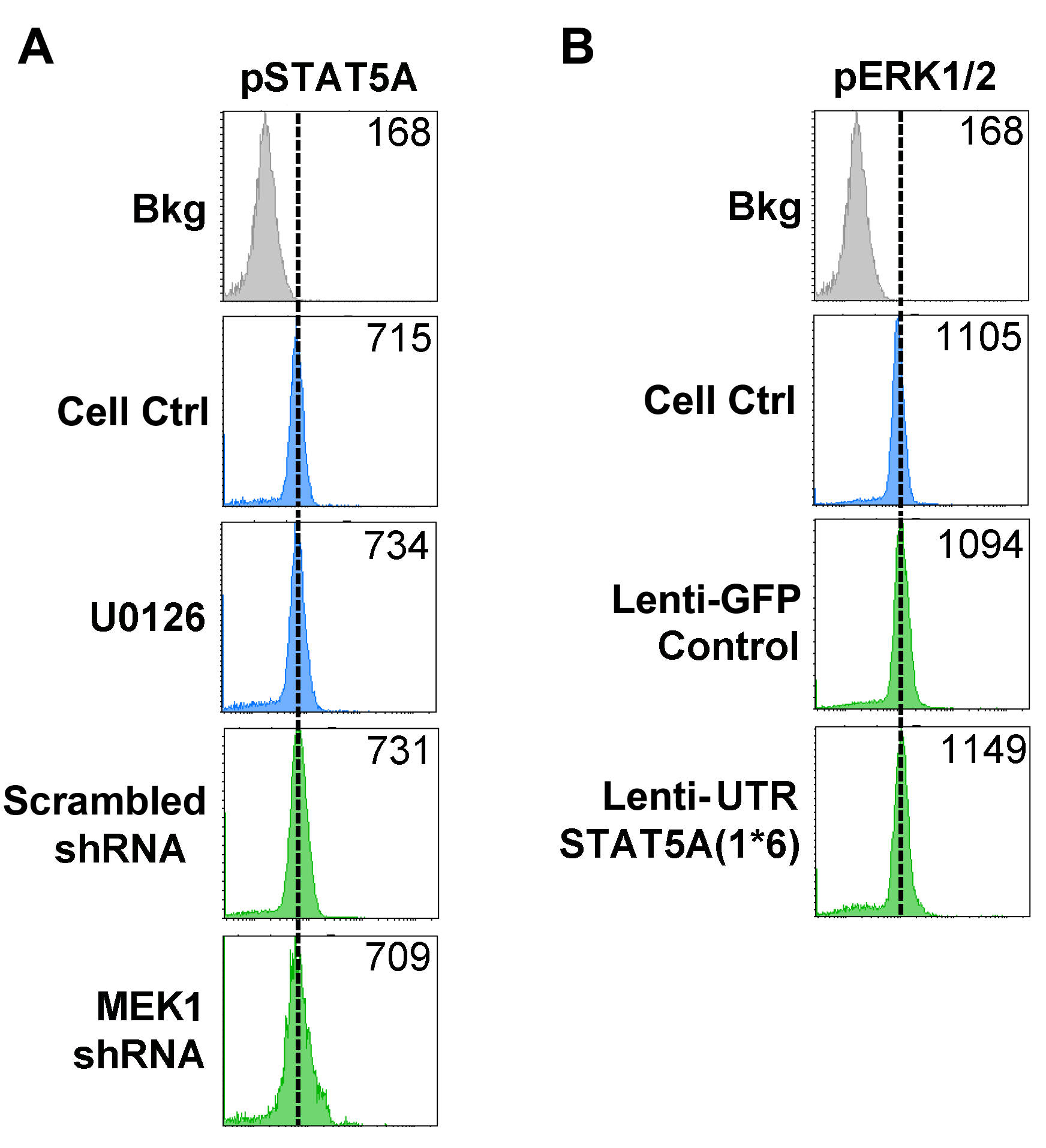

Supplement: Figure S5 — Phosphorylation of STAT5A and MEK does not affect each other significantly. (A) Day 7 CD36+ EPCs cultured under normoxia were either transduced with MEK-targeted shRNA (MEK shRNA) and scrambled shRNA, respectively, or treated with MEK-specific inhibitor U0126 at a final concentration of 10 µM. (B) Day 7 CD36+ EPCs cultured under normoxia were transduced with Lenti-GFP and Lenti-UTRSTAT5A(1*6). At 48 hrs post-transduction or post-treatment, the levels of pSTAT5A (A) and pERK1/2 (B) were analyzed by flow cytometry. The GFP-positive population of lentivirus-transduced cells was selectively gated. Numbers shown are MFI of the whole peak. Dashed reference line is selected arbitrarily to show the relative position of the peaks. Bkg, background, secondary antibody only. (TIF) [file ppat.1002088.s005.tif]

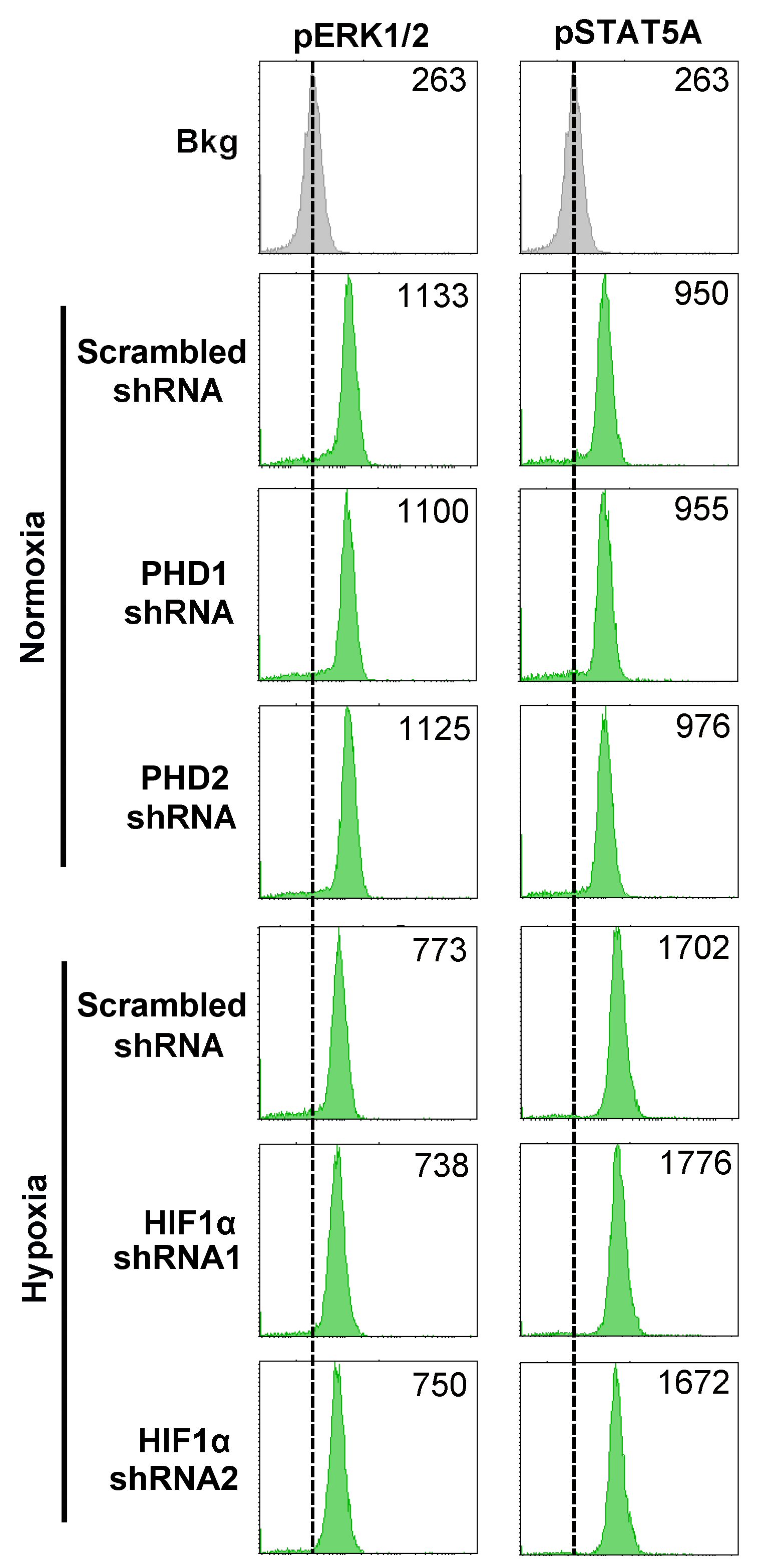

Supplement: Figure S6 — HIFα does not modulate phosphorylation of STAT5A and MEK1/2 under normoxia and hypoxia. Day 7 CD36+ EPCs cultured under either normoxia or hypoxia were transduced by respective shRNA-encoding lentivirus. The levels of phosphorylated ERK1/2 (pERK1/2) and phosphorylated STAT5A (pSTAT5A) were determined by flow cytometry at 48 hrs post-transduction. The dash line was drawn arbitrarily to show the shift of staining peak from the background control (Bkg). The numbers in the plots of the left and right columns show MFI values of pERK1/2 and pSTAT5A, respectively. (TIF) [file ppat.1002088.s006.tif]
