# Supplementary material for: Productive Parvovirus B19 Infection of Primary Human Erythroid Progenitor Cells at Hypoxia Is Regulated by STAT5A and MEK Signaling but not HIFα
Source: PLoS Pathog. 2011 Jun 16;7(6):e1002088. doi: 10.1371/journal.ppat.1002088 (PMC3116823; doi:10.1371/journal.ppat.1002088)
Supplement: Table S1 — Sequences of shRNAs used in this study. (DOC) [file ppat.1002088.s007.doc]

**Table S1. Sequences of shRNAs used in this study.**

| Name | Target of shRNA | shRNA sequence |
| --- | --- | --- |
| HIF1α  shRNA1 | HIF1α | 5’CGCTGGAGACACAATCATATCTCGAGATATGATTGTGTCTCCAGCGGTTTTT3’ |
| HIF1α  shRNA2 | HIF1α | 5’CAGTTATGATTGTGAAGTTACTCGAGTAACTTCACAATCATAACTGGTTTTT3’ |
| PHD1  shRNA | HIF PHD1 | 5’CGCATGGCAGACAGCTTAAATCTCGAGATTTAAGCTGTCTGCCATGCGTTTTT3’ |
| PHD2  shRNA | HIF PHD2 | 5’CTGTTATCTAGCTGAGTTCATCTCGAGATGAACTCAGCTAGATAACAGTTTTT3’ |
| PHD3  shRNA | HIF PHD3 | 5’CACCTGCATCTACTATCTGAACTCGAGTTCAGATAGTAGATGCAGGTGTTTTT3’ |
| STAT5A shRNA1 | STAT5A | 5’GACCATGTACTCGATCAGGATCTCGAGATCCTGATCGAGTACATGGTCTTTTT3’ |
| STAT5A  shRNA2 | STAT5A | 5’CGCTTTAGTGACTCAGAAATCTCGAGATTTCTGAGTCACTAAAGCGCTTTTT3’ |
| MEK1  shRNA | MEK1 | 5’GCTTCTATGGTGCGTTCTACACTCGAGTGTAGAACGCACCATAGAAGCTTTTT3’ |
| MEK2  shRNA | MEK2 | 5’CTTCCAGGAGTTTGTCAATAACTCGAGTTATTGACAAACTCCTGGAAGTTTTTT3’ |
| HIF2α  shRNA | HIF2α | 5’GGTGGAGCTAACAGGACATACTCGAGTATGTCCTGTTAGCTCCACCTTTTTT3’ |
| HIF3α  shRNA | HIF3α | 5’GCAGTGGAGACAGATTTAGATCTCGAGATCTAAATCTGTCTCCACTGCTTTTT3’ |
| p110α shRNA1 | PI3K p110α | TTCCTGATCTTCCTCGTGCTGCTCGAGCAGCACGAGGAAGATCAGGAATTTTTT |
| p110α  shRNA2 | PI3K p110α | GGCTGGGACCCGATGCGGTTACTCGAGTAACCGCATCGGGTCCCAGCCTTTT |

All the shRNA sequences listed above were designed based on the validated shRNA sequences from Sigma.
