# Supplementary material for: Productive Parvovirus B19 Infection of Primary Human Erythroid Progenitor Cells at Hypoxia Is Regulated by STAT5A and MEK Signaling but not HIFα
Source: PLoS Pathog. 2011 Jun 16;7(6):e1002088. doi: 10.1371/journal.ppat.1002088 (PMC3116823; doi:10.1371/journal.ppat.1002088)
Supplement: Text S1 — Supplemental methods. (DOC) [file ppat.1002088.s008.doc]

**Text S1. Supplemental Methods**

**Material and Methods**

**Cell viability assay:** We examined cell viability using CellTiter-Glo® kit (Promega), which determines the number of viable cells in culture based on quantification of the ATP presence in cells following the manufacturer’s instructions.

**Cell cycle analysis:** Cells were washed with PBS and fixed in 1% paraformaldehyde for 30 min. After being washed, the cells were then suspended in a staining buffer containing 1 µg/ml DAPI (4',6-diamidino-2-phenylindole), 0.3% Tween-20, and 2% FCS in PBS. Stained cells were analyzed on the 3-laser flow cytometer (LSR II; BD Biosciences).
